# Seasonal Shifts in Trophic Interaction Strength Drive Stability of Natural Food Webs

**DOI:** 10.1111/ele.70075

**Published:** 2025-02-01

**Authors:** Ursula Gaedke, Xiaoxiao Li, Christian Guill, Lia Hemerik, Peter C. de Ruiter

**Affiliations:** ^1^ Institute of Biochemistry and Biology University of Potsdam Potsdam Germany; ^2^ School of Ecology, Environment and Resources Guangdong University of Technology Guangzhou China; ^3^ Biometris, Department of Mathematical and Statistical Methods Wageningen University Wageningen The Netherlands; ^4^ Institute for Biodiversity and Ecosystem Dynamics University of Amsterdam Amsterdam The Netherlands

**Keywords:** asymptotic stability, diversity and stability, energetic feasibility, food web structure, long‐term data, mass‐balanced network, maximum loop weight, pelagic food web, seasonal and interannual dynamics, trophic interaction loops

## Abstract

It remains challenging to understand why natural food webs are remarkably stable despite highly variable environmental factors and population densities. We investigated the dynamics in the structure and stability of Lake Constance's pelagic food web using 7 years of high‐frequency observations of biomasses and production, leading to 59 seasonally resolved quantitative food web descriptions. We assessed the dynamics in asymptotic food web stability through maximum loop weight, which revealed underlying stability mechanisms. Maximum loop weight showed a recurrent seasonal pattern with a consistently high stability despite pronounced dynamics in biomasses, fluxes and productivity. This stability resulted from seasonal rewiring of the food web, driven by energetic constraints within loops and their embedding into food web structure. The stabilising restructuring emerged from counter‐acting effects of metabolic activity and competitiveness/susceptibility to predation within a diverse grazer community on loop weight. This underscores the role of functional diversity in promoting food web stability.

## Graphical abstract

We analysed the stability of Lake Constance's pelagic food web, which showed a recurring seasonal pattern and remained high despite pronounced variability in biomasses and energy flows. Stability was maintained through seasonal rewiring of the food web, driven by energetic constraints within interaction loops and their connection to incoming and outgoing flows (=‘openness’). This stabilising rewiring was supported by a functionally diverse grazer community, underscoring the role of functional diversity in promoting food web stability.

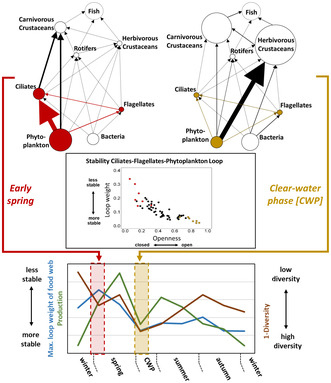

## 1 Introduction

**FIGURE 1:**
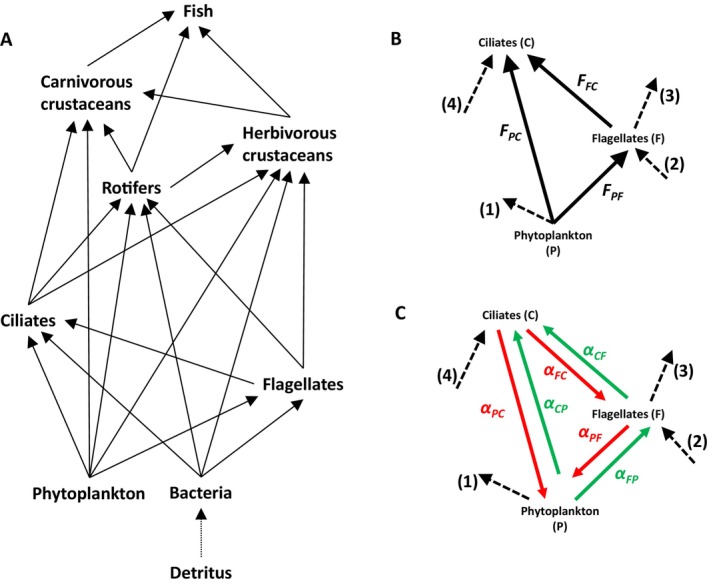
(A) Diagram of the Lake Constance food web. Combining measured biomasses and production rates with mass‐balance modelling provided estimates of 59 quantitative representations of the Lake Constance food web at up to nine different phases per year. (B) Example of a trophic interaction loop. Solid arrows: Fluxes inside the loop; dashed arrows numbered (1) to (4) represent fluxes going into or leaving the loop. There is no ingoing flux to phytoplankton as it is autotroph and we do not consider the flux leaving the ciliates because it does not affect fluxes in the loop. (C) Each flux in a loop involves two interaction strengths (green—positive, red—negative), for example, *α*_PC_ = −*F*_PC_/*B*_C_ and *α*_CP_ = *e*_C_ * F_PC_/B_P_. Together the six interaction strengths create two trophic interaction loops: A positive loop arising from the positive effect of phytoplankton on ciliates and the negative effects of ciliates on flagellates and of flagellates on phytoplankton (interactions *α*_CP_‐*α*_FC_‐*α*_PF_), and a negative loop going in the opposite direction, including two positive effects (of phytoplankton on flagellates and of flagellates on ciliates) and one negative effect (of ciliates on phytoplankton) (interactions *α*_FP_‐*α*_CF_‐*α*_PC_).

Biological communities in ecosystems worldwide are mostly characterised by high levels of species richness and large fluctuations in species abundances. In particular, within years in temperate regions, strong dynamics of mostly small, short living organisms may arise from variability in abiotic and biotic forcing. Nevertheless, the resulting seasonal patterns are frequently recurrent across years despite the multitude of influential factors and their pronounced variation (Kaartinen and Roslin 2012; Sommer et al. 2012; Vallina et al. 2023). Such recurrence suggests a sort of regulation that prevents major long‐term shifts in biomasses and species composition. A challenge addressed here is to identify and understand mechanisms that prevent long‐term shifts despite pronounced short‐term fluctuations. We analysed the stability of a highly dynamic community, that is, the pelagic food web of Lake Constance, based on comprehensive long‐term data, comprising 7 years of temporally highly resolved observations of the dynamics of all key groups of pelagic organisms (Boit and Gaedke 2014; Gaedke, Hochstädter, and Straile 2002) (Figure 1). The data reveal strong seasonal variability in biomasses and fluxes together with a remarkable boundedness over years (Gaedke, Hochstädter, and Straile 2002; Straile 1998) (Appendices S1 and S2). We focused on how the network of trophic interactions may indeed act as a stabilising agent. Trophic interactions are crucial for the persistence of species as they determine food availability for survival and the risk of extinction by overgrazing. Food web stability is therefore a key to understand population and community dynamics (Jacquet et al. 2016). Thus, we investigated whether, and if so, how and why food web stability varied over seasons and years, by adopting the loop weight approach (Neutel, Heesterbeek, and de Ruiter 2002).

Trophic interaction loops are pathways of trophic interactions starting and ending with one guild without passing other guilds more than once (Figure 1B,C). Loop weight is the geometric mean of the absolute values of the interaction strengths in the loop. Loops with an even number of negative links generate positive feedbacks and are therefore named positive (feedback) loops. The weight of the positive loops is particularly relevant as the positive feedbacks may destabilise food webs. For example, increasing phytoplankton biomass may lead to more ciliates, causing a higher grazing pressure on flagellates, reducing their biomass and grazing on the phytoplankton, which then, in turn, leads to even more phytoplankton biomass (Figure 1). Hence, the stronger the interaction strengths in such self‐enforcing loops, the higher the loop weight and the stronger the destabilising effect. Therefore, the maximum weight of any loops in the food web is proposed as an indicator of stability (Neutel, Heesterbeek, and de Ruiter 2002).

The loop weight approach enabled us to analyse biotic and abiotic drivers and their dynamics that affect maximum loop weight, and hence food web stability. Main drivers were seasonality in primary production, the metabolic rates of the trophic guilds in the heaviest loop, and how this loop is embedded into the food web structure as a whole. That is, we extended the consideration of energetic constraints from the level of individual guilds (Li et al. 2021) to the level of individual loops and the interactions the guilds in the loop have with guilds outside the loop. To quantify this embedding, we introduced a new measure called ‘openness’ which depends on the ratio between the fluxes going in or out of the loop (the four broken arrows in Figure 1B) and the sum of all fluxes entering or leaving the respective guilds. Hence, by looking at the dynamics in food web stability we obtained a mechanistic understanding of how dynamics in biomasses and fluxes are controlled, preventing long‐term shifts in food web structure and composition.

Finally, we examined whether our data may support some well‐known concepts regarding food web stability, that is, (1) the destabilising effect of enhanced primary productivity (Rosenzweig 1971), (2) that trophic interactions representing minor energy fluxes may strongly impact food web stability (McCann, Hastings, and Huxel 1998; Paine 1980), (3) that food web stability is positively correlated with large predator–prey body‐size ratios (Brose, Williams, and Martinez 2006) and (4) that a diversity in trophic pathways (cf. Appendix S1) may buffer population dynamics and enhance food web stability (MacArthur 1955).

## 2 Material and Methods

Lake Constance is a large (472 km^2^), deep (on average 101 m) lake situated north of the European Alps. Given its small littoral zone, large water volume (almost 50 km^3^) and mesotrophic state during the study period (1987–1993), it has often served as a model system for large open water bodies, limnetic or marine. Species were assigned to eight trophic guilds sharing the same prey and predators, that is, phytoplankton, bacteria, heterotrophic flagellates, ciliates, rotifers, predominantly herbivorous crustaceans, predominantly carnivorous crustaceans and zooplanktivorous fish (Figure 1). Feeding relationships were established based on diet information (Gaedke, Hochstädter, and Straile 2002). Additionally, we included a non‐living compartment that accounts for the particulate and dissolved organic matter originating from excretion and exudation and serves as food source for bacteria. It was considered in the mass balance modelling, but not in the Jacobian matrices which only included the strengths of the trophic interactions among the living trophic guilds.

Plankton was sampled weekly during the season and twice a month in winter at different depths. Abundances and body sizes were assessed by microscopy which enabled to calculate biomasses in terms of carbon using guild‐specific conversion factors (Gaedke 1992; Gaedke, Hochstädter, and Straile 2002). The production of phytoplankton (^14^C‐fixation), bacteria (^14^C‐leucine incorporation) and heterotrophic flagellates (dilution technique) was directly measured in situ (Tilzer et al. 1991; Simon and Rosenstock 1992; Weisse 1997). The production of the other plankton guilds was estimated by applying laboratory‐based, guild‐, temperature‐ and size‐specific growth rates to the measured abundances and size structure (Gaedke, Hochstädter, and Straile 2002). Mean annual fish biomass and production were estimated from Lake Constance catch data and sonar data (Boit and Gaedke 2014) (for details for all methods see https://fred.igb‐berlin.de/Lakebase).

To track seasonal dynamics, each year was subdivided into 7–9 phases (cf. Appendix S1). To reduce the impact of inter‐annual climatic variability, the beginning and end of most phases, lasting between 14 and 102 days and comprising 2–12 sampling dates, were not fixed to certain calendric dates but determined according to numerous physical, chemical and biological parameters for each year (Appendix—Table S2, Gaedke, Hochstädter, and Straile 2002). Phases 3 and 6 did not occur in 1988, and phase 5 not in 1990 and 1993.

Fluxes among guilds were calculated using mass‐balance modelling (Hart et al. 1997) based on the measured production and sedimentation rates and guild‐specific carbon: phosphorous ratios (Hochstädter 2000), and estimates of exudation, diet compositions and maximum trophic transfer efficiencies. We established in total 59 mass‐balanced food webs in units of C (surrogate for food quantity, energy) and P (surrogate for food quality) for each phase by averaging biomasses and fluxes across all sampling dates within each phase and considering temporal changes in biomasses over adjacent phases (Gaedke, Hochstädter, and Straile 2002). Here we used the food web descriptions in units of C.

The empirical quantitative mass‐balanced food webs directly provide values of the fluxes (*F*_*ij*_, mgC m^−2^ day^−1^), biomasses (*B*_*i*_, mgC m^−2^) and energy conversion efficiencies (*e*_*i*_) between consumer and prey guilds enabling to calculate interaction strength:

(1) $$\alpha_{\mathrm{ij}}=-\frac{F_{\mathrm{ij}}}{B_{j}}$$

for the negative effect of consumer *j* on prey *i*, and

(2) $$\alpha_{\mathrm{ji}}=e_{j}\frac{F_{\mathrm{ij}}}{B_{i}}$$

for the positive effect of prey *i* on consumer *j* (de Ruiter, Neutel, and Moore 1995), following the Lotka‐Volterra approach of May (1972) and Pimm (1982).

We consider the linear, asymptotic stability of the food webs as formally determined by the real part of the leading eigenvalue, *Re*(*λ*_max_), of their respective Jacobian matrices. This approach is only strictly valid for systems in equilibrium, a condition that is not fully met in our case as exogenous and endogenous processes alter the biomasses and fluxes over time. However, we argue that the biomass changes during individual phases are typically small compared to the fluxes among the guilds and that therefore the Jacobian matrices still reliably inform about the stability of the food webs (Appendix S3).

The empirical interaction strengths, Equations (1) and (2), are the off‐diagonal elements of the Jacobian matrices. The diagonal elements cannot be determined empirically and following for example, Jacquet et al. (2016), were therefore set to zero. This implies that *Re*(*λ*_max_) was always positive and could only be interpreted as the required strength of intra‐guild competition to render the food web stable (Tang, Pawar, and Allesina 2014). To circumvent this limitation, we used the loop‐weight approach by Neutel, Heesterbeek, and de Ruiter (2002), which provides maximum loop weight, *LW*_max_, as indicator for asymptotic food‐web stability, where larger loop weights represent less stable systems. Loop weights were calculated using only the empirically estimated off‐diagonal elements of the Jacobian matrices. By identifying the guilds that form the loop with the maximum weight, the approach further allows to identify combinations of guilds that are important for stability. The seasonal and interannual patterns of *Re*(*λ*_max_) and *LW*_max_ are highly similar (Appendix S4), the two measures were statistically significantly correlated (Spearman rank correlation *r*_S_ = 0.78, *p* < 0.001).

The weight of a loop, *LW*, is calculated as the geometric mean of the interaction strengths in the loop. For a positive loop of length three this gives:

(3) $$\;\mathrm{LW}=\sqrt[{3}]{\left|\alpha_{\mathrm{bi}}\right|\left|\alpha_{\mathrm{it}}\right|\left|\alpha_{\mathrm{tb}}\right|}$$

where the subscripts *b*, *i* and *t* refer to respectively the primary (=basic) resource, intermediate consumer and top predator in the loop (cf. Appendix S3). Following equation (1) and (2) *LW* is calculated via:

(4) $$\mathrm{LW}=\sqrt[{3}]{\frac{F_{\mathrm{bi}}}{B_{i}}\;\frac{F_{\mathrm{it}}}{B_{t}}e_{t}\frac{\;F_{\mathrm{bt}}\;}{B_{b}\;}}$$

Also guilds outside a loop can indirectly influence its weight, for example, by grazing on guilds in the loop or by being grazed by guilds in the loop. We calculated the strength of these influences in terms of the ‘openness’ of the loop. For a loop of length 3 starting with the primary resource (Figure 1B) we distinguished four in‐ and outgoing fluxes and defined their openness (*O*_*i*_ for flux *i* (=1,2,3,4)) by taking this ratio: (sum of all fluxes that go out of (arrows 1 and 3), or in (arrows 2 and 4) a considered trophic guild (*G*) but are not part of the loop (=*F*_*G‐O*_) divided by (sum of all fluxes at the considered trophic)).

For example, for flux (1) at the guild phytoplankton (Figure 1B,C), we considered the five fluxes outgoing from phytoplankton (Figure 1) and took the total of all fluxes from phytoplankton (*P*) to guilds outside the loop (*F*_*P–O*_), namely the three fluxes from phytoplankton to rotifers *F*_*P–R*_, to herbivorous crustaceans *F*_*P–Ch*_, and to carnivorous crustaceans *F*_*P–Cc*_, for example, $F_{P-O}=F_{P-R}+F_{P-\mathrm{Ch}}+F_{P-\mathrm{Cc}}$. For the denominator we added the two fluxes from phytoplankton within the loop *F*_*P–L*_, that is, the flux from phytoplankton to flagellates *F*_*P–F*_, and the one to ciliates *F*_*P–C*_. We then calculated the openness of the loop with respect to the first flux (*O*_*1*_) associated to phytoplankton as the ratio:

(5) $$O_{1}=\frac{F_{P-O}}{\sum_{j\in \left\{C,\mathrm{Cc},\mathrm{Ch},F,R\right\}}F_{P-j}}$$

Hence, *O*_1_ represents the fraction of primary production that leaves the loop compared to the total primary production. The three other types of openness (*O*_2_, *O*_3_ and *O*_4_) were calculated accordingly (cf. Appendix S5).

In addition to *O*_i_ quantifying how open a loop is at every guild and in‐ or outgoing flux, we calculated a measure of overall openness (*O*_o_) of the loop. We first defined the complement of each of the four openness measures (*O*_1_ *–O*_4_) as one minus the considered openness (*O*_1_ *–O*_4_). Then their geometric mean provides a measure of ‘closedness’, which gives equal weight to all four in‐ or outgoing fluxes independent of their quantitative importance and its complement one minus the geometric mean is the ‘mean’ openness of the loop:

(6) $$O_{o}=1-\sqrt[{4}]{\left(1-O_{1}\right)\left(1-O_{2}\right)\left(1-O_{3}\right)\left(1-O_{4}\right)}$$

## 3 Results

**FIGURE 2:**
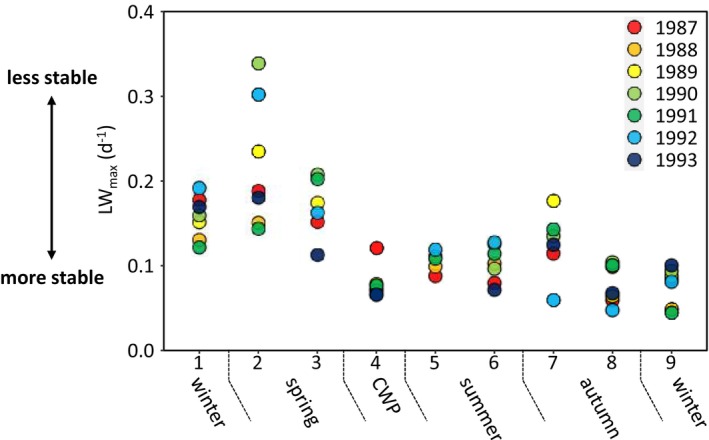
Maximum weight of all loops (LW_max_, d^−1^) with length 3 per seasonal phase over 7 years; colours denote year of observation. CWP stands for clear‐water phase, a period of intensive top‐down control of small plankters. We calculated the weights of all loops of all lengths and found that in only three cases the heaviest loops were longer than 3, surpassing maximum loop weight of the loops with length 3 only by 6%–11%. We therefore restricted the analysis to the 25 positive feedback loops with length 3 and calculated their maximum weight in the 59 food webs.

Based on comprehensive data from Lake Constance we analysed 59 quantitative mass‐balanced food web models covering up to nine different seasonal phases during 7 years of observation (Gaedke, Hochstädter, and Straile 2002). Biomasses, productions, fluxes, interaction strengths and thus food web structure showed large seasonal fluctuations, which followed a recurrent pattern over years (Boit and Gaedke 2014; Gaedke, Hochstädter, and Straile 2002) (Appendices S1 and S2). In line with previous studies, frequency distributions of interaction strengths were highly skewed (Appendix S2). To understand the long‐term boundedness despite pronounced short‐term fluctuations we analysed the stability of the Lake Constance food web using the loop weight approach. The loop with the maximum weight indicates food web stability in a negative sense, that is, the heavier the loop the less stable the food web. Maximum loop weight (*LW*_max_) exhibited a pronounced seasonality (Figure 2). In late winter (phase 1) *LW*_max_ was consistently at an intermediate level (stability intermediate), during early spring (phase 2) *LW*_max_ increased on average with a relatively high interannual variability (stability decreased). This was followed by a decrease of *LW*_max_ in late spring (phase 3) and a consistently low minimum value during a period of severe top‐down control on small organisms like phytoplankton, flagellates and ciliates by crustaceans, called the clear‐water phase (phase 4, maximum level of stability). Thereafter, *LW*_max_ slightly increased during summer (phases 5–7) and subsequently gradually declined during autumn and early winter (phases 8–9) (Figure 2).

**FIGURE 3:**
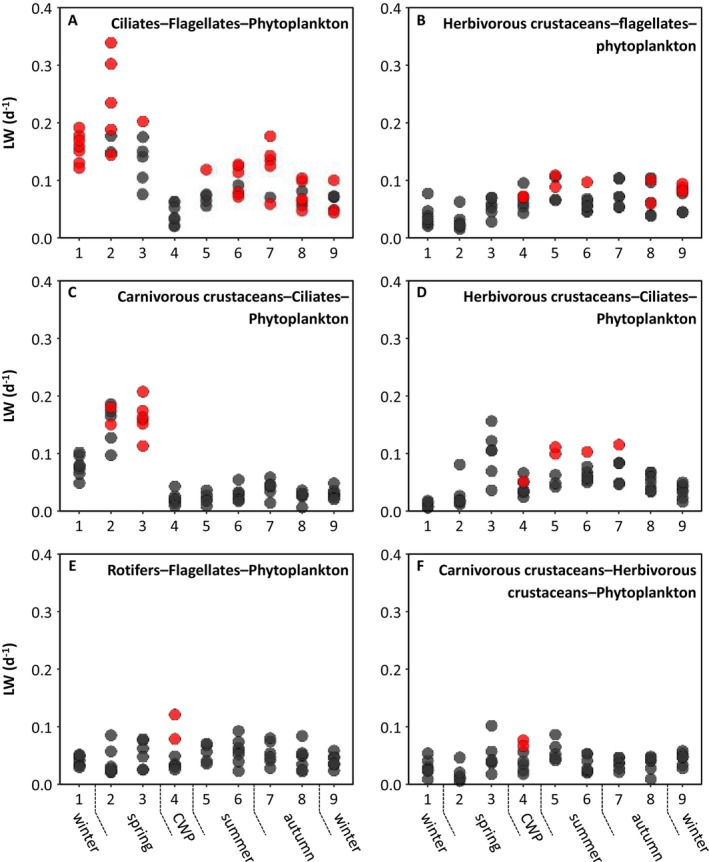
Weight of the six loops that were the heaviest in particular phases and years. Red dots denote that in these cases the loop was the heaviest. CWP stands for clear‐water phase.

We determined which trophic guilds were part of the (positive) loop with the maximum weight and how the composition of the loop with the maximum weight changed over time. Six different loops were the heaviest in the different phases and years (Table S1). The ciliates‐flagellates‐phytoplankton loop was the heaviest in 32 of the 59 webs, followed by the herbivorous crustaceans‐flagellates‐phytoplankton loop (in 11 webs), the carnivorous crustaceans‐ciliates‐phytoplankton loop (in seven webs), the herbivorous crustaceans‐ciliates‐phytoplankton loop (in five webs), the rotifers‐flagellates‐phytoplankton loop (in two webs) and the carnivorous crustaceans‐herbivorous crustaceans‐phytoplankton loop (in two webs) (Figure 3). The absolute values and seasonal dynamics in loop weight of these six loops differed remarkably. The largest maximum loop weights were found for the ciliates‐flagellates‐phytoplankton loop and the carnivorous crustaceans‐ciliates‐phytoplankton loop. The other loops became the heaviest when these loops, and the ciliates‐flagellates‐phytoplankton loop in particular, had relatively low weights (Figure 3). Remarkably, considering the entire annual cycle, stability tended to be low during phases when usually the same loop was the heaviest whereas stability was higher during phases with a large interannual variability in the loops becoming the heaviest (Appendix S6). This suggests that a diversity of potential loops achieving similar weights enhances stability.

Subsequently, we investigated why a particular loop was the heaviest during a particular phase and year. We looked into metabolic rates of the guilds in the loop, and how the loop was embedded into the food web. Loop weight is positively related to the energy conversion efficiency of the top predator in the loop and the flux/biomass ratios of the three guilds in the loop (cf. Equation (4)). This helps to explain the composition of the loop with the maximum weight:

1. Flux/biomass ratios were the largest for the fastest growing guilds promoting loop weight. In the Lake Constance food web these were the smallest guilds, that is, phytoplankton, heterotrophic flagellates and ciliates, with average biomass‐specific production rates (determining flux/biomass ratios) of 0.60, 0.47 and 0.15 day^−1^, respectively. For the other guilds production rates were 0.12 day^−1^ (bacteria), 0.12 day^−1^ (rotifers), 0.04 day^−1^ (herbivorous crustaceans) and 0.07 day^−1^ (carnivorous crustaceans). They explain (i) why phytoplankton was always the primary resource in the heaviest loop, (ii) why almost always heterotrophic flagellates or ciliates were the intermediate consumer, and ciliates often the top predator in the heaviest loop; the only exception was the carnivorous crustaceans‐herbivorous crustaceans‐phytoplankton loop that was twice the heaviest in 59 webs, and (iii) why the ciliates‐flagellates‐phytoplankton loop was most frequently the heaviest loop.
2. Loop weight also depended on ‘openness’, that is, the fluxes leaving the loop to or entering the loop from guilds outside the loop. In loops of length 3 there are four types of ‘openness’ fluxes (Figure 1B, Equation (5)), which we expected to influence the flux/biomass ratios in the following way:
  - When a large proportion of the production of the primary resource goes to guilds outside the loop (*O*_1_, dashed arrow 1 in Figure 1B), then the fluxes to both consumers in the loop will be small compared to the biomass of the primary resource. This will decrease the interaction strengths among the primary resource and the consumers and hence loop weight (cf. Equation (4)). In analogy, loop weight is lowered when a large proportion of the production of the intermediate consumer goes to guilds outside the loop (*O*_3_, dashed arrow 3 in Figure 1B), as this will decrease the interaction strength between the intermediate consumer and the top predator.
  - When the intermediate consumer feeds strongly on guilds outside the loop (*O*_2_, dashed arrow 2 in Figure 1B), consumption from the primary resource will be small relative to its biomass, which will decrease the interaction strength between the primary resource and the intermediate consumer and hence loop weight. The same holds when the top predator predates substantially on guilds outside the loop (*O*_4_, dashed arrow 4 in Figure 1B).

**FIGURE 4:**
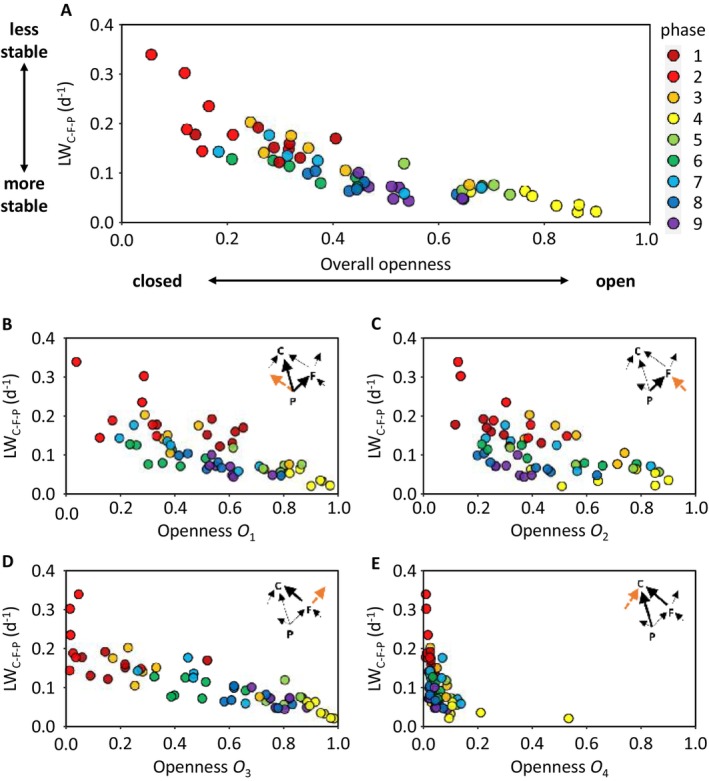
(A) Relationship between the weight of the ciliates‐flagellates‐phytoplankton loop, LW_C‐F‐P_, and its overall openness (*O*_*o*_) which was calculated as the geometric mean of the four types of openness (*O*_*1*_ *‐O*_*4*_): $O_{o}=1-\sqrt[{4}]{\left(1-O_{1}\right)\left(1-O_{2}\right)\left(1-O_{3}\right)(1-O_{4)}}$ (Spearman correlation coefficient *r*_S_ = −0.90, *p* < 0.001). (B–E) Correlation between the weight of the ciliates‐flagellates‐phytoplankton loop and the four individual types of openness calculated as the ratios between the size of an in‐ or outgoing flux into a guild (marked in red in the inserted food web sketches) and the sum of all fluxes within or outside the loop of the respective guild (see Methods and Materials) (*O*_1_: *r*_S_ = −0.76, *O*_2_: *r*_S_ = −0.60, *O*_3_: *r*_S_ = −0.88, *O*_4_: *r*_S_ = −0.68). Colours denote seasonal phases.

To see whether these mechanisms were apparent in the Lake Constance food web, we correlated the weight of the ciliates‐flagellates‐phytoplankton loop, *LW*_*C‐F‐P*_, that is, the loop that was most frequently the heaviest, with overall openness and the four types of openness calculated for all 59 food webs (i.e. independently of whether the ciliates‐flagellates‐phytoplankton loop was the heaviest). As expected, *LW*_*C‐F‐P*_ was negatively correlated with overall openness (stronger than in random webs, Appendix S7) and three of its components, *O*_1_, *O*_2_ and *O*_3_ (Figure 4). *O*_4_ was always small as ciliates consume little prey from outside the loop. *O*_1_–*O*_4_ correlated slightly more with *LW*_*C‐F‐P*_ than with the individual interaction strengths since they were not independent from each other (Appendix S5).

These findings hold for the other loops as well, for example, their loop weight was also negatively correlated with overall openness (Appendix S7). Some scatter in the relationship between overall openness and loop weight arises from differences in the trophic structure of the loop, that is, whether most of the primary resource production is channelled to the intermediate consumer or directly to the top predator (Appendix S7 and S8). In the first case, loop weight is high at a given openness as the two negative interaction strengths are comparably high whereas in the second case only the positive interaction strength is promoted.

**FIGURE 5:**
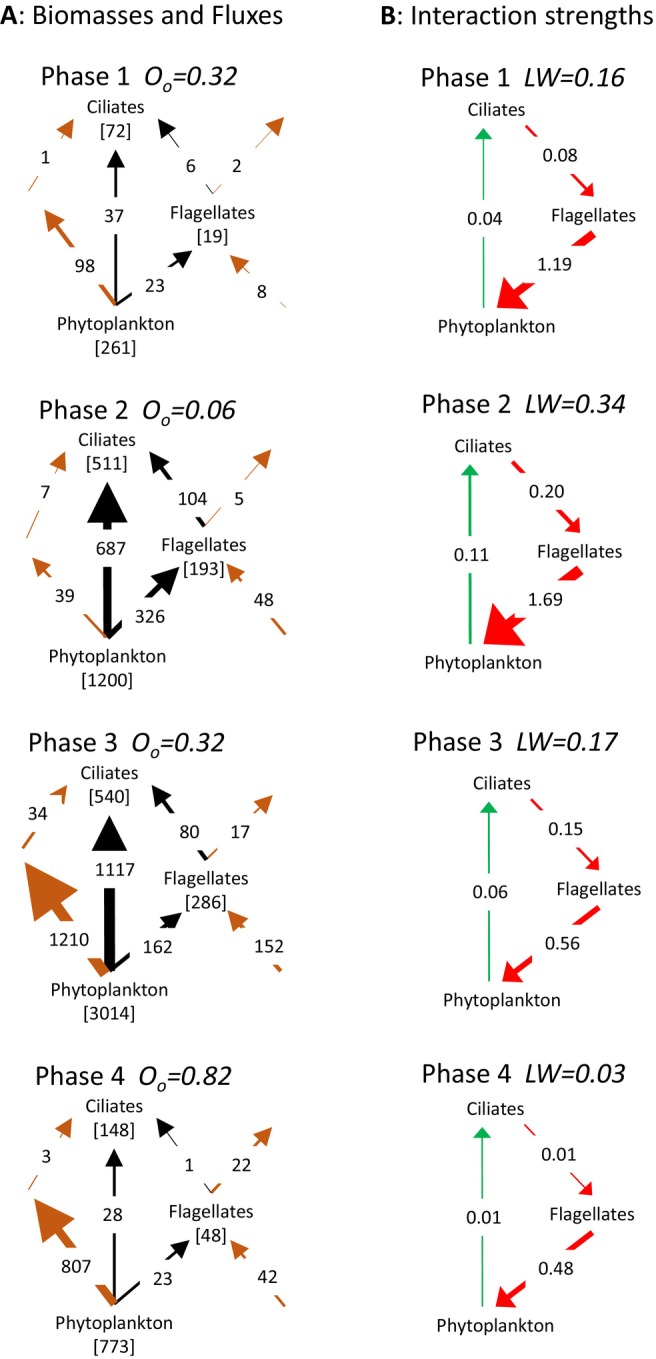
Dynamics in the distributions of biomasses, fluxes, interaction strengths and overall openness *O*_O_ in the ciliates‐flagellates‐phytoplankton loop, and the consequences for loop weight, *LW*, during the first four phases in 1990. Numbers along the arrows denote fluxes in mg C m^−2^ d^−1^ (A) or absolute interaction strengths in d^−1^ (B). Numbers between brackets denote biomasses in mg C m^−2^ (A). The figure illustrates how the loop became first more closed as small flagellates and ciliates benefitted faster from increasing phytoplankton production but were then outcompeted or grazed by larger consumers. Hence, the loop became more open which reduced loop weight and hence enhanced stability. In phase 1 and 2 in 1990 the ciliates‐flagellates‐phytoplankton loop was the heaviest one. In phase 3, the carnivorous crustaceans‐ciliates‐phytoplankton loop was the heaviest and in phase 4 the herbivorous crustaceans‐flagellates‐phytoplankton loop.

To better understand the distinct seasonal succession in the type of the heaviest loop (Figure 3), which strongly influenced stability, Figure 5 shows how the weight of the ciliates‐flagellates‐phytoplankton loop changed with its openness during the first 4 phases in 1990. From late winter (phase 1) to early spring (phase 2), phytoplankton production increased almost 7‐fold as result of increasing light, temperature and stratification (Tirok and Gaedke 2007). The effect on the fluxes was stronger than on the biomasses, which increased interaction strengths and loop weight as the smaller organisms responded faster than the larger organisms and bacteria. Hence, most phytoplankton production was consumed within the ciliates‐flagellates‐phytoplankton loop and its consumers received little food from outside. This strongly reduced the openness from 0.32 to 0.06 and enhanced loop weight. From phase 2–3 phytoplankton production further increased approximately 2.5‐fold, but the destabilisation that occurred between phases 1 and 2 was halted by alterations in the food web structure: flagellates relied more on bacteria and phytoplankton was more grazed by the larger crustaceans (Figure 5, Appendix S1). This re‐increased openness to 0.32 and reduced loop weight. The consequence was that the carnivorous crustaceans‐ciliates‐phytoplankton loop became the heaviest at this phase in 5 out of 6 years (Table S1). From phase 3–4 competition and grazing by larger guilds further increased leading to low biomasses of phytoplankton, flagellates and ciliates and reduced the fluxes and interaction strengths among them, even furthering the opening of their loop to 0.82 and reducing its weight.

In summary, the ciliates‐flagellates‐phytoplankton loop comprised the three metabolically most active guilds which thus had the highest flux/biomass ratios, rendering it the most likely candidate for being the heaviest loop. However, during parts of the season larger guilds in the food web competed with or fed on its three component guilds, thereby opening this loop to such an extent that its weight declined below the weight of other loops despite the lower weight‐specific metabolic rates of guilds in these loops. For example, in phase 4 and 5, crustaceans strongly suppressed the smaller guilds and often became the top predators in the heaviest loop despite their comparably low weight‐specific metabolic rates (Figure 3), resulting in low maximum loop weights. These low loop weights coincided with a top‐heavy biomass distribution and large predator–prey biomass and body weight ratios.

## 4 Discussion

In Lake Constance and many other habitats, dynamics in biomasses and production, in particular those of small short living organisms, are characterised by strong yet restricted seasonal fluctuations and a relative constancy over years (Gaedke, Hochstädter, and Straile 2002). This boundedness may partly arise from intra‐guild competition generating density‐dependent regulation (for Lake Constance phytoplankton and ciliates see for example, Tirok and Gaedke 2007). Such boundedness may also arise from direct and indirect effects of the trophic interactions in the food web (McCann, Hastings, and Huxel 1998). Thus, we related the observed recurrent seasonal pattern to the dynamics in the weight and composition of the trophic interaction loops that generated destabilising positive feedbacks. This loop weight approach revealed that stability changed seasonally, mostly driven by the metabolic activity of the guilds in the heaviest loop and by loop openness, that is, how potentially heavy loops were embedded in the food web context.

The Lake Constance dataset had sufficient temporal resolution and replicates (7 years) to study seasonal dynamics and interannual recurrence in food web structure and allowed to directly calculate food web stability using the loop weight approach (Neutel, Heesterbeek, and de Ruiter 2002). The use of the maximum loop weight was justified by its strong correlation with asymptotic stability (Appendices S4, S6, S9, cf. Li et al. 2021; Neutel and Thorne 2014; Neutel, Heesterbeek, and de Ruiter 2002).

Beyond providing a measure of food web stability, the loop weight approach enabled a mechanistic understanding of how food web stability was the outcome of the interplay between (1) physiological properties of the trophic guilds, (2) the trophic structure within the loops, (3) how biomasses and fluxes inside loops were constrained by energetic feasibility, (4) the strength of the interactions between guilds inside the loop compared to those with guilds outside the loop, that is, openness, and (5) seasonal changes in environmental conditions.

The biomass‐specific metabolic rates of the guilds influence the flux/biomass ratios and thus loop weight. These were the largest for small, fast growing guilds, that is, phytoplankton, heterotrophic flagellates and ciliates. Hence, the loop including these guilds was potentially the heaviest, phytoplankton was always the primary resource in the heaviest loop, and flagellates or ciliates occurred in all except two heaviest loops.

Second, with everything else being equal, loop weight was higher when within the loop the flux from the primary resource to the intermediate consumer was larger than the flux to the top predator, as the resulting increase of the two negative interaction strengths has a stronger effect on loop weight than the related weakening of the positive interaction strength (Appendix S8).

A third, important factor constraining loop weight was the energetic feasibility within loops (Li et al. 2021). For example, if one consumer ingests a large fraction of the primary resource, little is left for the other consumer. Similarly, if the top predator receives a large fraction of its diet from the primary resource, consumption on the intermediate consumer will be low. Such energetic constraints create ‘compensatory effects’ among the interaction strengths within a loop, which restricts maximum loop weight and herewith enhances stability (Li et al. 2021). This mechanism was clearly supported by our data (Appendix S9).

Fourth, we showed that energetic constraints act also at the food web level and strongly enhance stability as they determine openness. The larger the fluxes going in or out of a loop, the lower loop weight, as these fluxes lower the internal fluxes and thus the flux/biomass ratios within the loop. Thus, openness and hence stability depend on guilds outside the loop, providing alternative resources or imposing predation pressure on loop members. Hence, the food web context in which potentially heavy loops are embedded is highly relevant for overall food web stability (compare with random webs lacking these constraints, Appendix S7).

Fifth, dynamics in food web stability coincided with dynamics in environmental processes. In early spring, growth conditions improved for all guilds, but the small phytoplankton, flagellates and ciliates increased faster than larger crustaceans and bacteria. This reduced the openness and increased the weight of the heaviest loop, mostly consisting of the three smallest guilds, leading to the least stable food webs. Subsequently, the effect of improved growth conditions was counteracted by an altered food web structure. During late spring, phytoplankton production continued to increase but now a larger proportion went to guilds outside the ciliates‐flagellates‐phytoplankton loop, in particular to the crustaceans. This reduced the weight of this loop to such an extent that other loops became the heaviest ones (except 1991), albeit on a lower absolute level given the lower flux/biomass ratios of the trophic guilds in these loops. This stabilising effect of openness was most pronounced during the clear‐water phase when stability was maximal although the production/biomass ratio of phytoplankton was maximal (Rocha et al. 2011). During this phase, we encountered the highest interannual variability in the loops being the heaviest due to the tight balance in the counteracting interplay between metabolic rates of consumer guilds and openness. Over the 7 years of observation, four different loops were the heaviest in this phase and had similar weights, so that small environmental changes and sampling variability may have influenced which loop became the heaviest (for details see Appendix S1). To a lesser extent, this pattern prevailed during summer and early autumn, during which grazing pressure continued to reduce biomasses and fluxes of the smaller organisms.

Overall, the dynamics in the composition and weight of the heaviest loops reflect the counter‐acting effect (trade‐off) between high metabolic rates (promoting loop weight) and competitiveness/susceptibility to predation, promoting openness and thus reducing loop weight. That is, consumer guilds may be either small, metabolically active and vulnerable to competition and consumption by larger consumers outside the loop, or they are large and may monopolise the consumption of the primary resource by outcompeting or predating on smaller consumers. This reduces openness but loop weight remains nevertheless restricted due to their low flux/biomass ratios. Such trade‐offs between maximum growth rate (r‐strategists) and competitiveness/defence (K‐strategists) promote ongoing shifts between them as none is perfect in all respects but each has its moment of opportunity during the season when it performs best. The trade‐offs and their positive effect on consumer diversity are widely established (Reich 2014; Wright et al. 2004; Züst and Agrawal 2017), including the plankton community in Lake Constance (Ehrlich, Kath, and Gaedke 2020; Ehrlich and Gaedke 2020). Hence, if metabolically highly active guilds dominate in a loop, they give rise to high interaction strengths but they are also susceptible to competition and predation, which opens the loop in the long run. This creates a negative feedback on loop weight promoting stability of diverse food webs. More generally, diversity in food webs may promote stability as biomasses and metabolic rates of guilds in self‐reinforcing positive feedback loops will only increase until predation or parasitism from outside the loop is strongly enhanced or essential resources are exploited, that is, openness and density‐dependent regulation via other food web components counteract the internal re‐enforcement. Our data confirmed such positive functional diversity‐stability relationship (Appendix S6) in line with Rooney et al. (2006).

We based our study on snapshots which reflect the truly existing types of webs at distinct times, instead of looking at long‐term (e.g. annually) averaged food webs which never exist at any point (McMeans et al. 2015) and are therefore less relevant to study. We accounted for the biomass changes from one snapshot (phase) to the next during the mass‐balancing. The observed seasonality in stability reveals that stability is not an inherent property of a food web in a distinct habitat but may change temporally as may the organisms decisive for stability. Albeit contributing only few percent to the community biomass, small, often undersampled organisms may be highly relevant for stability. The mechanisms we identified to drive stability add another facet how functional diversity influences stability. As they are not system‐specific, they may allow predicting the stability and changes thereof of other less studied food webs.

In addition, our results may provide empirical evidence for some well‐known concepts in food web theory. The first is the destabilising effect of increased primary productivity during spring as anticipated by the concept of the paradox of enrichment (Rosenzweig 1971). In our food webs, enhanced primary productivity generated high flux/biomass ratios and low openness within the loop with the fastest growing guilds and thus destabilising positive feedbacks. However, given our dynamic food web structure subsequently increasing grazing by larger crustaceans had a counteracting and hence stabilising effect via enhanced openness and lower flux/biomass ratios. This is in line with results of modelling studies showing that the introduction of higher trophic level guilds neutralised the destabilising effect of increased primary productivity (de Ruiter, Neutel, and Moore 2005; Neutel et al. 2007).

Furthermore, large predator–prey body weight ratios promoted food web stability in a model food web (Brose, Williams, and Martinez 2006). In Lake Constance, the body weight ratio between phytoplankton and their grazers, representing the decisive predator–prey interaction in this web, increased approximately 1000‐fold from early spring to the clear‐water phase due to the shift in the dominant herbivores from small ciliates to larger‐sized crustaceans (Boit and Gaedke 2014; Ehrlich and Gaedke 2020). This had a twofold effect on maximum loop weight. First, it opened the potentially heaviest loop formed by the small, metabolically most active guilds decreasing loop weight. Second, the loops that became the heaviest instead included larger herbivores with lower metabolic rates. Thus, their loop weight was also relatively low, implying high food web stability at high predator–prey weight ratios.

The observed effect of openness on stability may also help to explain why trophic interactions representing minor energy fluxes may have considerable impact on food web stability (McCann, Hastings, and Huxel 1998; Paine 1980, 1992). For example, grazing on flagellates by other guilds than ciliates represented only a minor flux in the food web as a whole (Figure 5, Appendix S1) but could strongly reduce the strength of the interaction between flagellates and ciliates and herewith the weight of the ciliates‐flagellates‐phytoplankton loop, being the most decisive one for stability (Figure 4D).

Finally, we found that a more even magnitude (and thus a higher diversity) of the fluxes from the primary producers to the different herbivorous guilds promoted food web stability (compare early spring with later phases during seasonal succession, Appendices S1 and S6). However, this effect was overruled during the brief clear‐water phase exhibiting maximum stability, which was characterised by a high top‐down control of small organisms by large herbivores, dominating community biomass and fluxes. This stabilisation by diverse trophic pathways provides an empirical example of the notion of MacArthur that complex ecological networks may buffer fluctuations in species abundances, and with this enhance food web stability (MacArthur 1955).

## Author Contributions

P.C.d.R. and U.G. designed the study, P.C.d.R. performed the research supported by U.G., C.G., L.H. and X.L., U.G. provided the data, their interpretation and new methods, and P.C.d.R. and U.G. wrote the manuscript supported by C.G., X.L. and L.H.

### Peer Review

The peer review history for this article is available at https://www.webofscience.com/api/gateway/wos/peer‐review/10.1111/ele.70075.

## Supporting information


**Appendix S1.** Supporting Information.

## Data Availability

The data supporting the results are provided under https://datadryad.org/stash/share/npC‐ikAFqp9j6O‐aSE6‐mBpcYHbytCOrCo‐fMZG15YQ.
