# Supplementary material for: Seasonal Shifts in Trophic Interaction Strength Drive Stability of Natural Food Webs
Source: Ecol Lett. 2025 Feb 1;28(1):e70075. doi: 10.1111/ele.70075 (PMC11786205; doi:10.1111/ele.70075)
Supplement: Supplementary file 1 — Appendix S1. Supporting Information. [file ELE-28-0-s001.pdf]

Appendix of

Seasonal shifts in trophic interaction strength drive stability of natural food webs

Ursula Gaedke, Xiaoxiao Li, Christian Guill, Lia Hemerik & Peter C. de Ruiter

Table of content

1. **Appendix Table S1** Composition of the loop with the maximum weight for each phase and year
2. **Appendix Table S2** Calendric dates for each phase and year
3. **Appendix S1** Seasonal dynamics in the food web structure revealed by quantitative food webs for each phase and information on the mass-balanced approach
4. **Appendix S2** Seasonal and interannual variability of the biomasses of the 7 major trophic groups, primary production, and mean negative and positive interaction strengths as well as the distributions of interaction strengths**.**
5. **Appendix S3** Underlying assumptions of the loop weight approach and their potential bearing on our results
6. **Appendix S4** Seasonal and interannual variability of Jacobian stability (Re(λ_{max})) and its correlation and temporal synchronisation with maximum loop weight
7. **Appendix S5** Calculation of the openness at individual guilds and their correlation with interaction strengths
8. **Appendix S6** Relationship between the diversity in the heaviest loops and stability
9. **Appendix S7** Relationship between overall openness and loop weight for the six loops, which were temporally the heaviest ones
10. **Appendix S8** Impact of the distribution of fluxes within the loop on loop weight
11. **Appendix S9** Energetic constraints within loops restrict maximum loop weight in mass-balanced food webs

Appendix Table S1 Composition of the loop with the maximum weight for each phase and year (not all phases occur in each year).

Year \ Phase	1	2	3	4	5	6	7	8	9
1987	Cil-HF-P	Cil-HF-P	CC-Cil-P	R-HF-P	HC-HF-P	Cil-HF-P	HC-Cil-P	HC-HF-P	HC-HF-P
1988	Cil-HF-P	CC-Cil-P		HC-HF-P	HC-Cil-P	HC-Cil-P		Cil-HF-P	Cil-HF-P
1989	Cil-HF-P	Cil-HF-P	CC-Cil-P	R-HF-P	HC-Cil-P	Cil-HF-P	Cil-HF-P	Cil-HF-P	HC-HF-P
1990	Cil-HF-P	Cil-HF-P	CC-Cil-P	HC-HF-P		HC-HF-P	Cil-HF-P	Cil-HF-P	HC-HF-P
1991	Cil-HF-P	Cil-HF-P	Cil-HF-P	CC-HC-P	HC-HF-P	Cil-HF-P	Cil-HF-P	HC-HF-P	Cil-HF-P
1992	Cil-HF-P	Cil-HF-P	CC-Cil-P	CC-HC-P	Cil-HF-P	Cil-HF-P	Cil-HF-P	Cil-HF-P	HC-HF-P
1993	Cil-HF-P	CC-Cil-P	CC-Cil-P	HC-Cil-P		Cil-HF-P	Cil-HF-P	Cil-HF-P	Cil-HF-P

Cil-HF-P	Ciliates – heterotrophic flagellates – phytoplankton
CC-Cil-P	Carnivorous crustaceans – ciliates – phytoplankton
R-HF-P	Rotifers – heterotrophic flagellates – phytoplankton
HC-HF-P	Herbivorous crustaceans – heterotrophic flagellates – phytoplankton
CC-HC-P	Carnivorous crustaceans – herbivorous crustaceans – phytoplankton
HC-Cil-P	Herbivorous crustaceans – ciliates – phytoplankton

Appendix Table S2 Calendric dates for each phase and year

Year \ Phase	1	2	3	4	5	6	7	8	9	end
**1987**	01/01	13/04	03/05	13/06	26/06	08/08	28/08	23/09	16/10	31/12
**1988**	01/01	10/04		28/05	11/06	10/07		10/09	08/11	31/12
**1989**	01/01	11/03	15/04	21/05	03/06	24/06	28/07	30/09	15/11	31/12
**1990**	01/01	16/03	22/04	13/05		10/06	31/08	29/09	30/10	31/12
**1991**	01/01	11/03	28/04	02/06	14/06	20/07	07/09	07/10	02/11	31/12
**1992**	01/01	03/03	11/04	31/05	20/06	19/07	29/08	04/10	11/11	31/12
**1993**	01/01	08/03	14/04	28/05		19/06	15/08	02/10	03/11	31/12

Starting dates of the 9 seasonal phases and the last date of phase 9. Otherwise, a phase ends a day before the next phase starts except for phase 5 in 1987, which ends on 25/07 and phase 2 in 1991, which ends on 06/04. The gaps until the next phases were omitted to improve the interannual comparability, as the conditions encountered during these periods did not occur in any other year. In 1987 a very unusual flooding event occurred, and in 1991 stratification patterns were unusually variable, i.e., neither unstratified nor stratified (not all phases occur in each year).

Appendix S1 Seasonal dynamics in the food web structure revealed by quantitative food webs for each phase and information on the mass-balanced approach

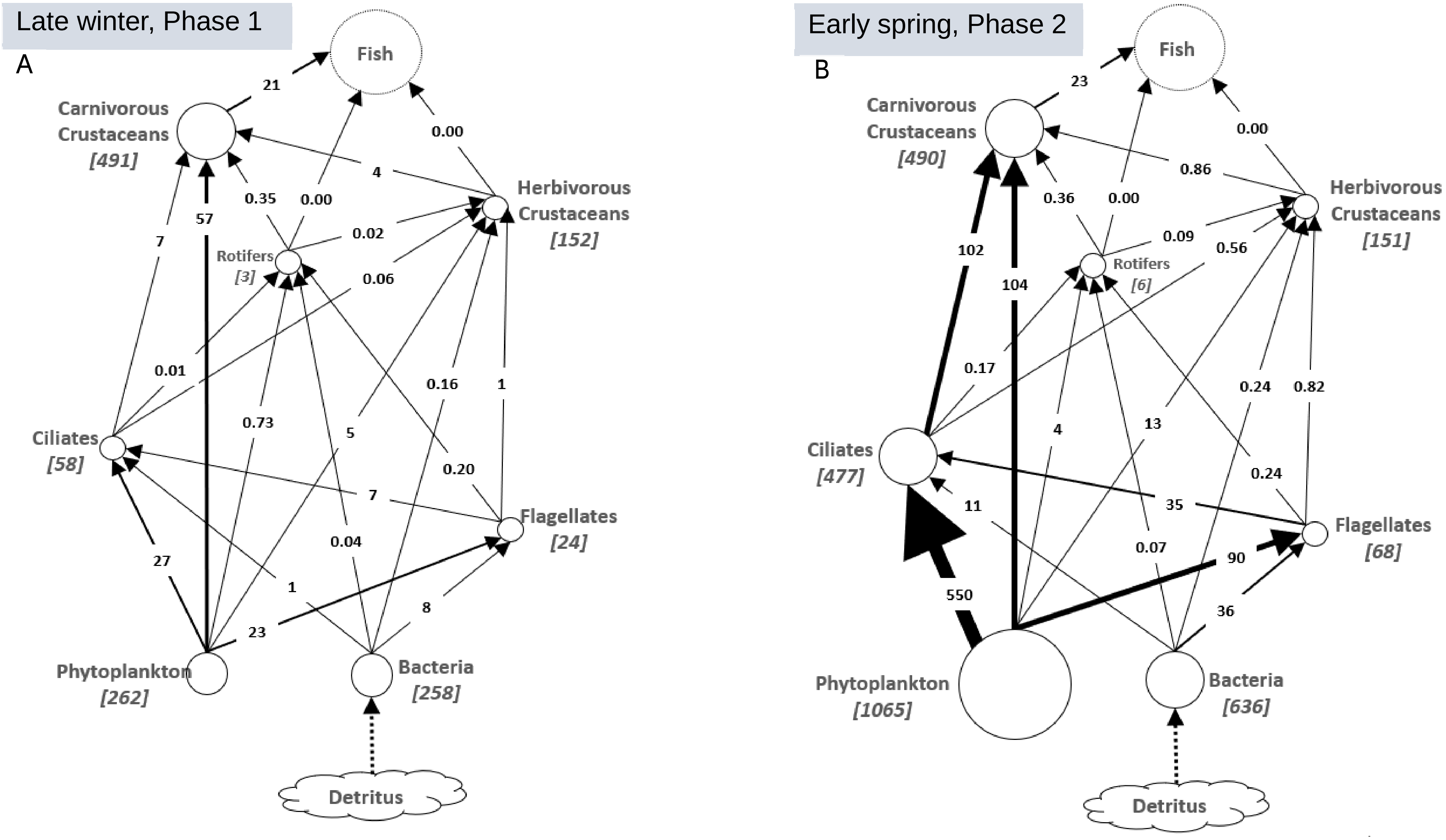

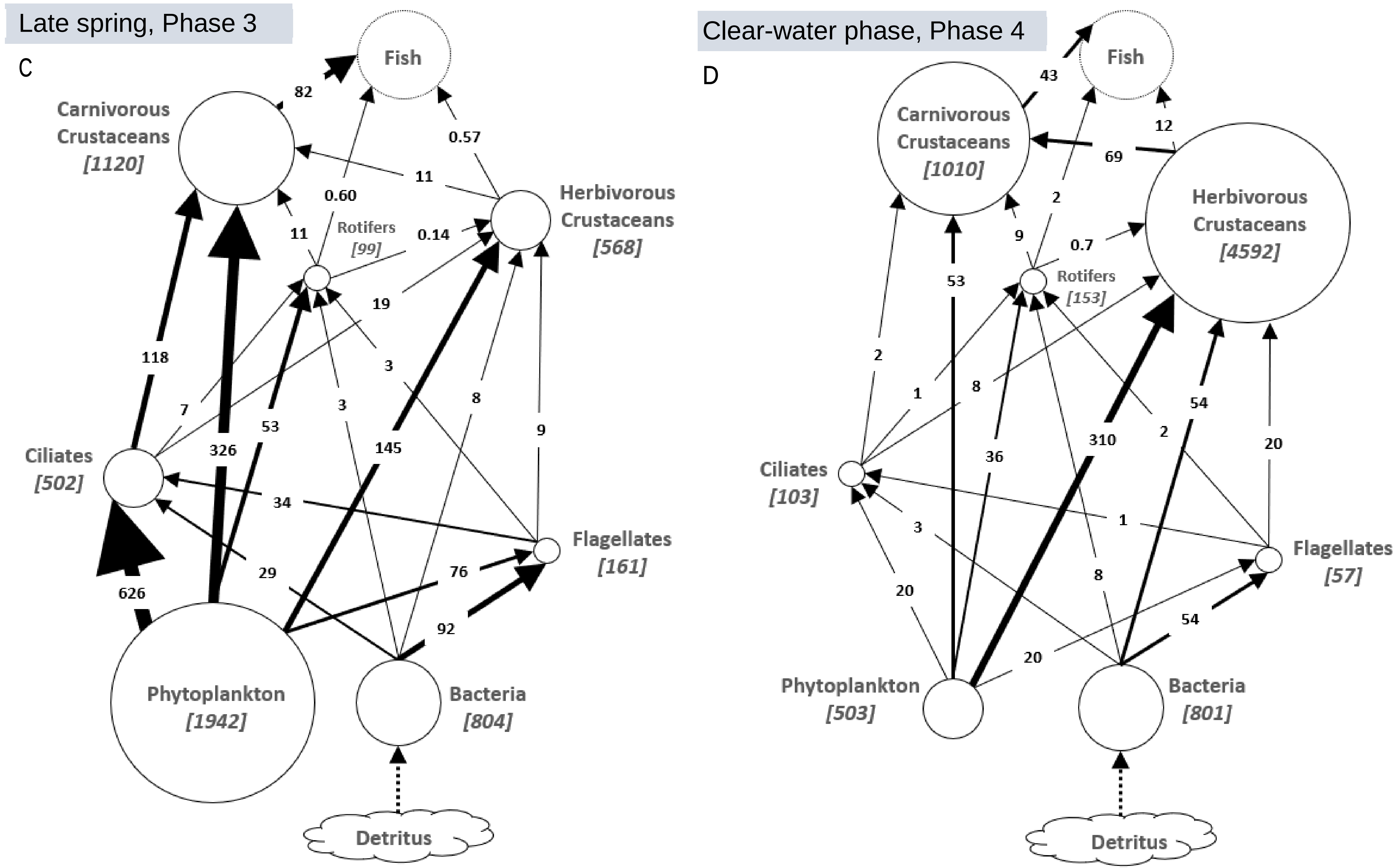

Late spring, Phase 3
C
Fish
82
Carnivorous Crustaceans [1120]
0.57
0.60
11
Herbivorous Crustaceans [568]
11
Rotifers [99]
0.14
19
118
7
3
3
8
53
9
Ciliates [502]
326
145
34
Flagellates [161]
29
76
626
92
Phytoplankton [1942]
Bacteria [804]
Detritus
Clear-water phase, Phase 4
D
Fish
43
12
Carnivorous Crustaceans [1010]
69
Herbivorous Crustaceans [4592]
2
9
0.7
53
Rotifers [153]
2
1
8
54
310
2
20
36
Ciliates [103]
3
1
Flagellates [57]
20
8
54
20
Phytoplankton [503]
Bacteria [801]
Detritus

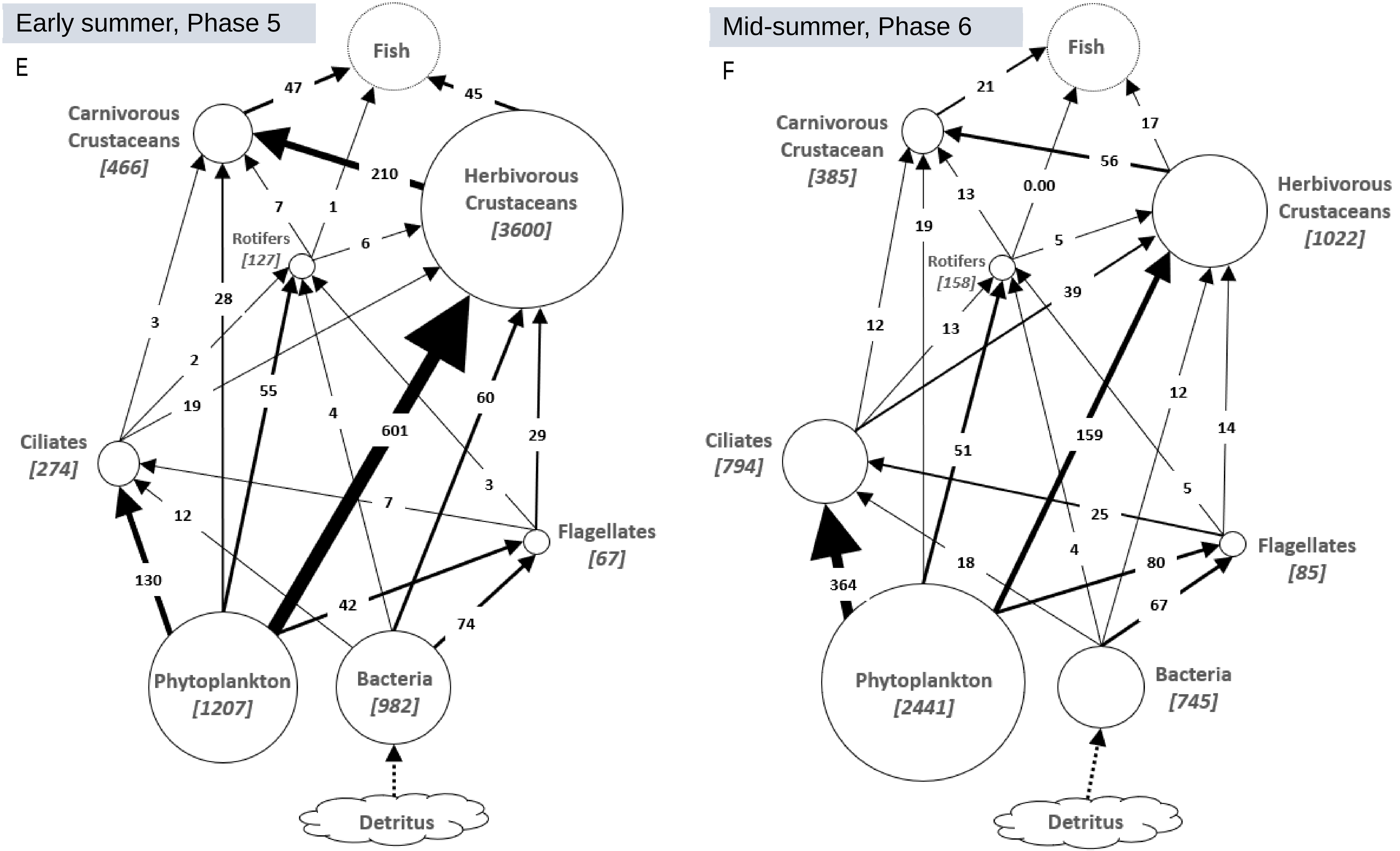

Early summer, Phase 5
E
Fish
47
45
Carnivorous Crustaceans [466]
210
Herbivorous Crustaceans [3600]
7
1
Rotifers [127]
6
3
28
2
19
55
4
601
60
29
Ciliates [274]
12
7
3
Flagellates [67]
130
42
74
Phytoplankton [1207]
Bacteria [982]
Detritus
Mid-summer, Phase 6
F
Fish
21
17
Carnivorous Crustacean [385]
56
Herbivorous Crustaceans [1022]
13
0.00
19
5
Rotifers [158]
39
12
13
12
14
Ciliates [794]
51
159
5
25
18
4
80
364
67
Flagellates [85]
Phytoplankton [2441]
Bacteria [745]
Detritus

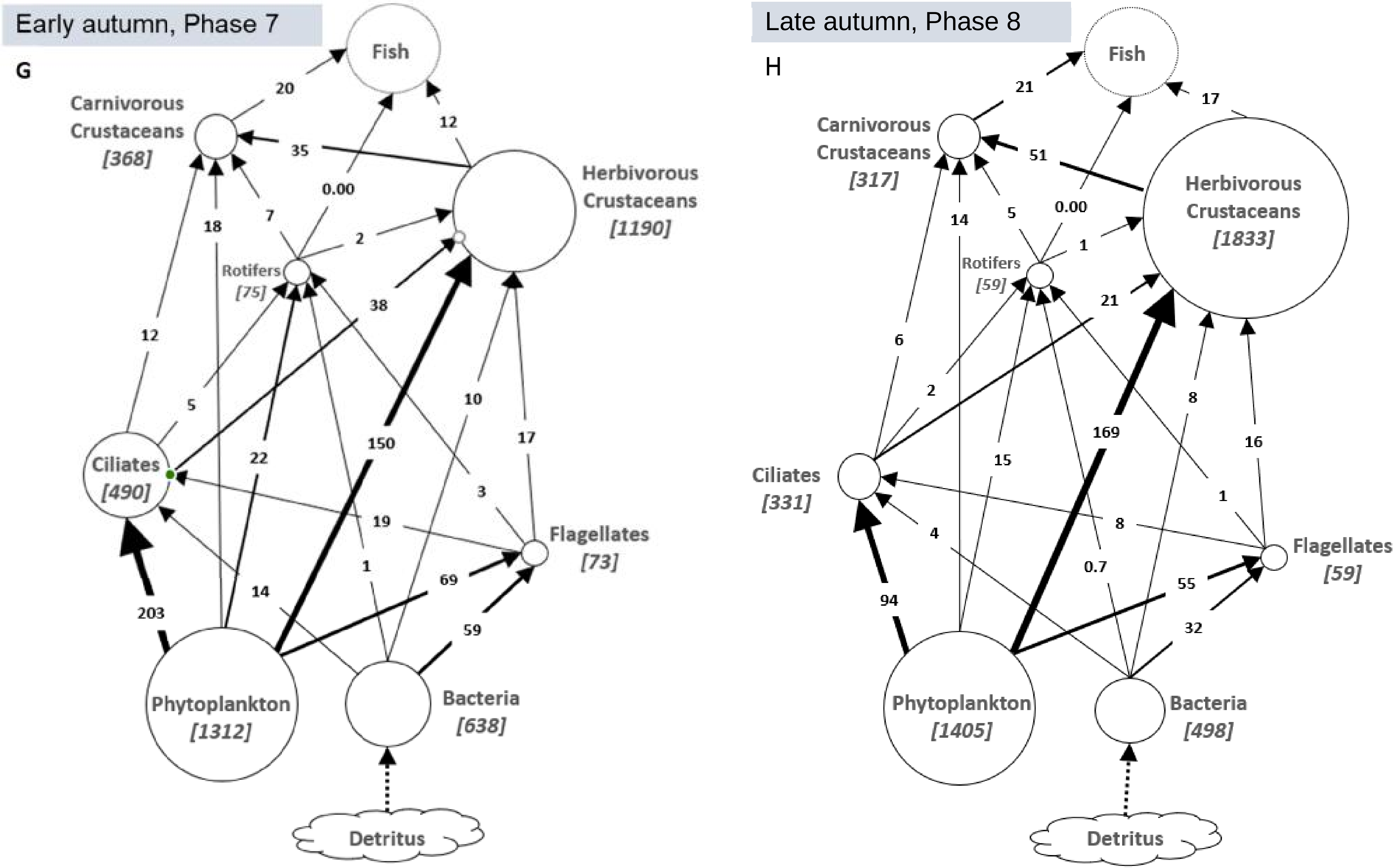
Early autumn, Phase 7
G
Fish
Carnivorous Crustaceans [368]
Herbivorous Crustaceans [1190]
Rotifers [75]
Ciliates [490]
Flagellates [73]
Phytoplankton [1312]
Bacteria [638]
Detritus
20
12
35
0.00
18
7
2
38
12
5
22
150
10
17
3
19
1
69
14
203
59
Late autumn, Phase 8
H
Fish
Carnivorous Crustaceans [317]
Herbivorous Crustaceans [1833]
Rotifers [59]
Ciliates [331]
Flagellates [59]
Phytoplankton [1405]
Bacteria [498]
Detritus
21
17
51
0.00
14
5
1
21
6
2
169
8
16
15
1
8
4
0.7
55
94
32

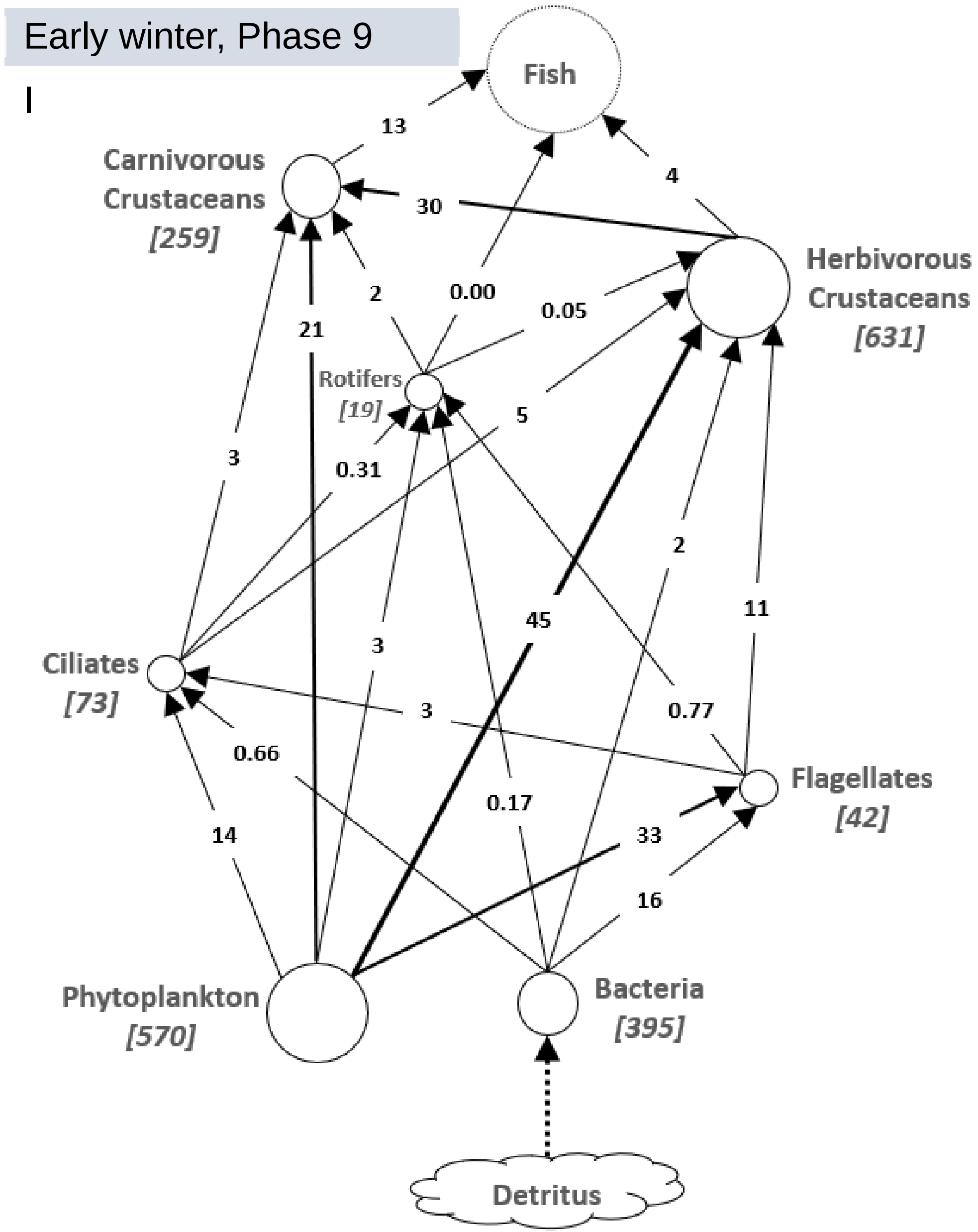

Appendix Figure 1 *Changes in biomasses and fluxes among the trophic guilds during the 9 seasonal phases. Numbers in the figure represent averages (back transformed after log_{10} transformation) of biomasses (mg C m^{-2}, numbers between brackets) and fluxes (mg C m^{-2} d^{-1}, numbers along arrows) per phase over the 7 years of observation. Values of 0.00 represent fluxes < 0.005 mg C^{-2} d^{-1}. That is, the binary food web structure does not change as all fluxes are present in each phase and year but the quantity is highly variable among fluxes and across time. As these numbers are averages across years, the figure pictures food web structures that were not exactly observed, but given the large variability between phases and the low variability between years, these averages can serve as representative for a particular phase.*

We distinguished the following phases, which were only partly defined by calendric dates but mostly according to the seasonal development of abiotic and biotic factors during the year under consideration (**Appendix Table S2**). For example, the onset of the clear-water phase varied between Mid-May and Mid-June depending on the previous weather conditions. Using such phase definitions reduced the impact of interannually varying weather conditions and improved our mechanistic

understanding of the seasonal development. Phase 1: Late Winter (starting 1st of January), Phase 2: Early Spring with first phytoplankton and protozoan development but still unstable stratification of the water column, Phase 3: Late Spring with stable stratification and higher temperatures promoting growth of crustaceans, Phase 4: Clear-Water Phase with strong competition for phytoplankton and a strong top-down control on phytoplankton, flagellates and ciliates by crustaceans, Phase 5: Recovery of the now often less edible phytoplankton and ciliates with still high biomasses of crustaceans, Phase 6: Mid-Summer with peaks of phytoplankton and ciliates, Phase 7: Late summer/early autumn with mostly declining biomasses, Phase 8: Autumn with some re-increase of phytoplankton and herbivorous crustaceans, and Phase 9: Early Winter with first deep mixing events and declining biomasses (ending 31st of December). In some years some phases did not occur: phases 3 and 6 in 1988, and phase 5 in 1990 and 1993 (for details see e.g., Boit & Gaedke 2014; Gaedke et al. 2002; Straile 1998; https://fred.igb-berlin.de/Lakebase).

Description of the abiotic growth conditions, major trophic interactions and reasons, why which loop became the heaviest:

A. In **phase 1** (late winter with low temperature, low light, deep mixing, and high nutrient concentrations) all biomasses and fluxes were low, and the maximum loop weight was at an intermediate level. The ciliates-flagellates-phytoplankton loop was the heaviest loop in all years, due to the high biomass-specific ingestion and production rates of these guilds.

B. In **phase 2** (early spring with still low temperature, increasing irradiance but still unstable stratification, high nutrient concentrations) most biomasses and the fluxes in particular went up. As a consequence, maximum loop weight increased. Compared to phase 1, a larger part of the phytoplankton and ciliate production went to the (juveniles of the) carnivorous crustaceans. The ciliates-flagellates-phytoplankton loop was the heaviest in 5 years. In two years, 1988 and 1993, the carnivorous crustaceans-ciliates-phytoplankton loop became the heaviest. In 1988 this was because the flux from flagellates to ciliates was small (28 mg C m^{-2} d^{-1}), decreasing the ciliates-flagellates-phytoplankton loop weight, while the flux from ciliates to carnivorous crustaceans was large (252 mg C m^{-2} d^{-1}). In 1993 this was because the flux from phytoplankton to flagellates was small (46 mg C m^{-2} d^{-1}), while the flux from phytoplankton to carnivorous crustaceans was large (281 mg C m^{-2} d^{-1}).

C. In **phase 3** (late spring with increasing temperature and light, stable stratification and onset of a moderate nutrient depletion) the tendency that fluxes increased more than the biomasses continued. However, this did not lead to a heavier weight of the ciliates-flagellates-phytoplankton loop because a larger proportion of the phytoplankton production went to the carnivorous crustaceans and to a lesser extent also to the herbivorous crustaceans. This increased the openness and thus decreased the weight of the ciliates-

flagellates-phytoplankton loop and made the carnivorous crustaceans-ciliates-phytoplankton loop the heaviest, except in 1991. In that year, the flux of phytoplankton to the carnivorous crustaceans was relatively small (209 mg C m^{-2} d^{-1}), while the fluxes from phytoplankton to flagellates (185 mg C m^{-2} d^{-1}) and from flagellates to ciliates (75 mg C m^{-2}) were comparably large, making the ciliates-flagellates-phytoplankton loop the heaviest. Compared to phase 2, the maximum loop weight often declined despite the raising temperature enhancing the weight-specific metabolic rates and thus the flux/biomass ratios, because the now ruling carnivorous crustaceans-ciliates-phytoplankton loop involved with the carnivorous crustaceans a much larger, rather slow growing top predator with substantially lower weight-specific metabolic rates.

D. In **phase 4** (a short period with still raising temperature and some nutrient recycling called clear-water phase because the biomass of phytoplankton and similar sized heterotrophs such as heterotrophic flagellates and ciliates is strongly reduced by a severe grazing pressure often predominantly imposed by the high biomass of herbivorous crustaceans such as daphnids) maximum loop weight was low in all years. Most fluxes were small, except the flux from phytoplankton to the herbivorous crustaceans. Therefore, this flux was part of the loop with the maximum weight in 5 years despite the low biomass-specific production rate of the herbivorous crustaceans compared to ciliates and also rotifers. In two years, 1987 and 1989, the rotifers-flagellates-phytoplankton loop was the heaviest. In both years, the flux from phytoplankton to the herbivorous crustaceans was small: 57 mg C m^{-2} d^{-1} in 1987 and 37 mg C m^{-2} d^{-1} in 1989. In 1987, the clear-water phase was late and less expressed since the biomass of herbivorous crustaceans was exceptionally low (1477 mg C m^{-2} d^{-1}) and that of rotifers high (777 mg C m^{-2} d^{-1}) due to an unusually cold winter and spring. Accordingly, all three fluxes in the rotifers-flagellates-phytoplankton loop were larger than average: from phytoplankton to flagellates 148 mg C m^{-2} d^{-1}, from phytoplankton to rotifers 241 mg C m^{-2} d^{-1} and from flagellates to rotifers 40 mg C m^{-2} d^{-1}. In phase 4, the ciliates-flagellates-phytoplankton loop was never the heaviest loop due to the severe grazing pressure by the crustaceans, making the loop very open (cf. Fig. 5 main text). In line, the mean maximum loop weight was at its minimum, due to the low biomass-specific metabolic rates of the top guilds, in particular the crustaceans and to a lesser extent the rotifers. Furthermore, given the low algal biomass the absolute phytoplankton production was low despite the maximal production/biomass ratio, leading to food shortage and thus low flux/biomass ratios of the herbivores.

E. In **phase 5** (early summer with a re-increase in primary production and recovery of now often defended and slower growing phytoplankton after the clear water phase, still high biomasses of herbivorous crustaceans, high temperature and increasing nutrient depletion) the flux from phytoplankton to the herbivorous crustaceans went further up, and also the

fluxes from phytoplankton to ciliates, and from herbivorous crustaceans to the carnivorous crustaceans. In 4 out of 5 years (phase 5 did not occur in 1990 and 1993) the large flux from phytoplankton to the herbivorous crustaceans was in the heaviest loop. In one year, 1992, the ciliates-flagellates-phytoplankton loop was the heaviest. In this year, the flux from phytoplankton to herbivorous crustaceans was small (290 mg C m^{-2} d^{-1}), reducing the weight of this loop. Maximum loop weight remained below the late winter-spring level.

F. In **phase 6** (mid-summer with a moderate phytoplankton bloom, maximum consumer and hence also flux diversity and the most severe nutrient depletion) the flux from phytoplankton to herbivorous crustaceans decreased and the flux from phytoplankton to ciliates became the largest. In 6 out of 7 years this flux was part of the heaviest loop. In 5 cases this was the ciliates-flagellates-phytoplankton loop and in one year, 1990, the herbivorous crustaceans-ciliates-phytoplankton loop. This was because in 1990 the flux from phytoplankton to ciliates was small whereas the flux from phytoplankton to the herbivorous crustaceans was large. Maximum loop weight remained again low compared to spring because of the low biomass specific production of the phytoplankton due to nutrient depletion and investment into grazing resistance, and the low metabolic rates resulting in low biomass-specific production of the large herbivorous crustaceans facing additionally suboptimal food conditions.

G. In **phase 7** (late summer/early autumn when solar irradiance and temperature start to decline and near surface mixing starts, slightly relaxing nutrient depletion) the ciliates-flagellates-phytoplankton loop was the heaviest loop, except in 1987 when the herbivorous crustaceans-ciliates-phytoplankton loop was the heaviest. The re-occurrence of the ciliates-flagellates-phytoplankton loop as the heaviest loop went along with an increase in mean maximum loop weight but not up to the spring level. This arises from a lower production/biomass ratio of phytoplankton (on average 0.35 d^{-1}, compared to e.g., phase 2 when it was on average 0.70 d^{-1}), food shortage of ciliates lowering ingestion and thus the flux/biomass ratio, and more open ciliates-flagellates-phytoplankton loops.

H. In **phase 8** (late autumn, decline in light, temperature and most of the biomasses while nutrient availability increases) the ciliates-flagellates-phytoplankton loop was the heaviest in 5 out of 7 years. In 1987 and 1991 instead of the ciliates, herbivorous crustaceans were the top consumer guild in the heaviest loop. In 1987, the flux from phytoplankton to ciliates was small (60 mg C m^{-2} d^{-1}), while the flux from phytoplankton to the herbivorous crustaceans was large (309 mg C m^{-2} d^{-1}).

I. In **phase 9** (early winter with low irradiance, low temperature and the first deep mixing events, increasing nutrient concentrations in the surface layer) all biomasses and fluxes were low and so was maximum loop weight. In 3 years (1988, 1991, 1993) the ciliates-

flagellates-phytoplankton loop was the heaviest, in the other 4 years (1987, 1989, 1990, 1992) the herbivorous crustaceans-flagellates-phytoplankton loop.

There are several options to derive interaction strengths from observational time-series data. We used the mass-balance approach for the following main reasons:

1. A major advantage of our data set is that it contains exceptionally strong measurements of the production at the base of the food web (primary production and bacterial production), which determines the activity of the higher trophic levels. The production data thereby allows estimating fluxes much more accurately than information that can be obtained from biomass dynamics. The mass-balance approach makes full use of this data. The ability to seamlessly account for process rates in the mass-balance approach also extends to further measurements such as sedimentation rates, which we included depending on the species composition of the phytoplankton.
2. The mass-balance approach enabled us to account for food quantity (measured in units of C) and food quality (measured in units of P) as we established mass-balanced webs in both commodities (Hart et al. 1997). When autotrophs get nutrient depleted, which happened in Lake Constance in summer, herbivores may be more limited by the availability of nutrients like P or N (needed to build up e.g., RNA or amino acids) than energy (C). We first established mass-balanced webs in units of C and then checked whether the supply of P was sufficient to enable the anticipated consumer production using the webs mass-balanced in P. If required, we reduced the production in units of C until the needs in both C and P were met. To obtain the webs in units of P we could use our direct measurements of C:P ratios of the individual groups. Thus, it was straightforward to include these two-fold constraints on the fluxes in the mass-balance approach.
3. Further advantages of the approach are that it enabled us to constrain the assimilation and growth efficiencies in C and P to realistic values, providing a good control that the algorithm delivered ecologically and physiologically realistic solutions. Additionally, the algorithm allowed us to verify that the calculated flux values led to plausible diet compositions and to systematically explore the consequences of distinct assumptions (e.g., concerning the maximum assimilation and growth efficiencies, cf. Hart et al., 1997; Gaedke et al. 2002) and of the sampling uncertainty for the overall food web structure.

Appendix S2 Seasonal and interannual variability of the biomasses of the 7 major groups, primary production, and mean negative and positive interaction strengths as well as the distributions of interaction strengths.

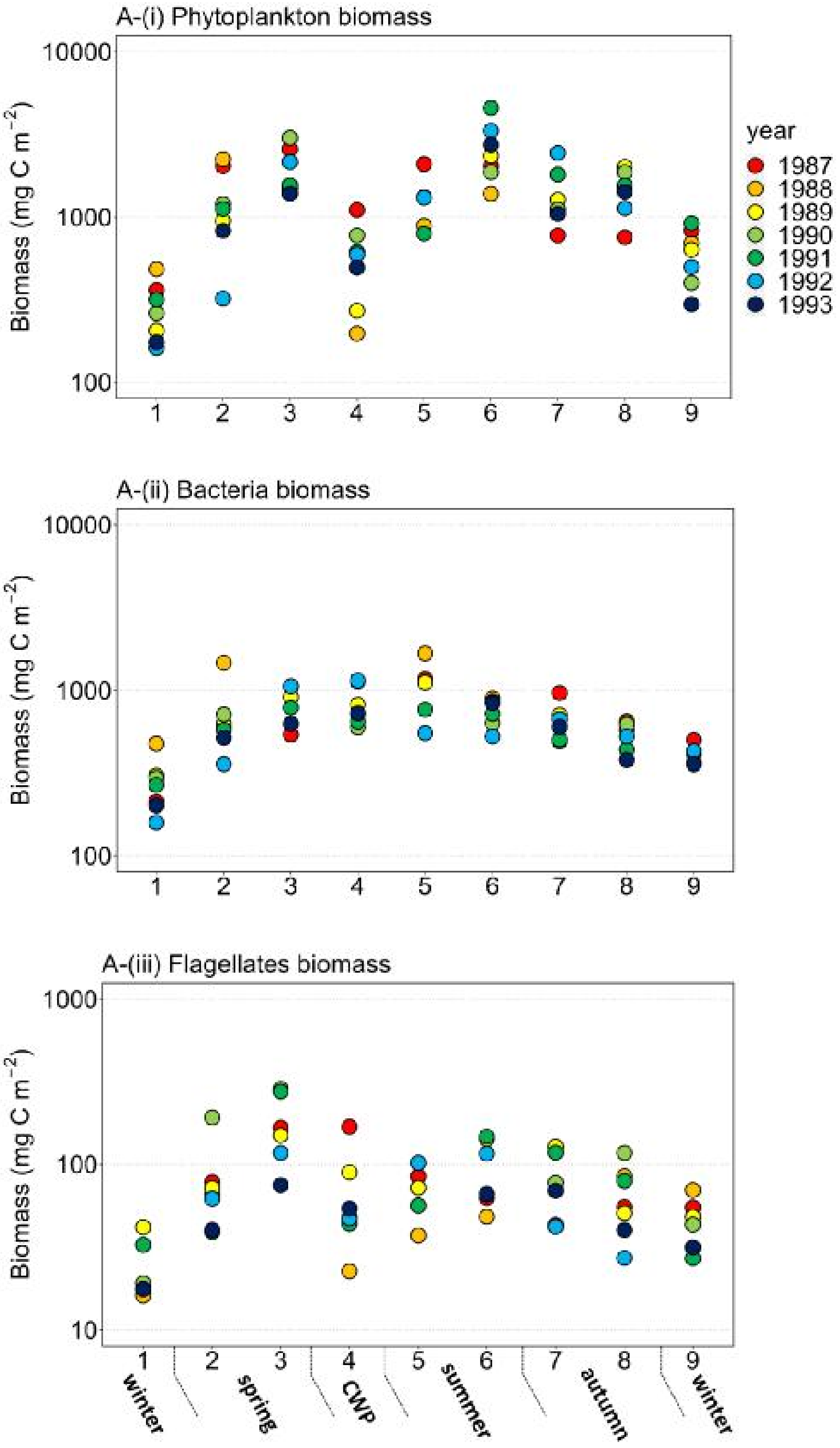

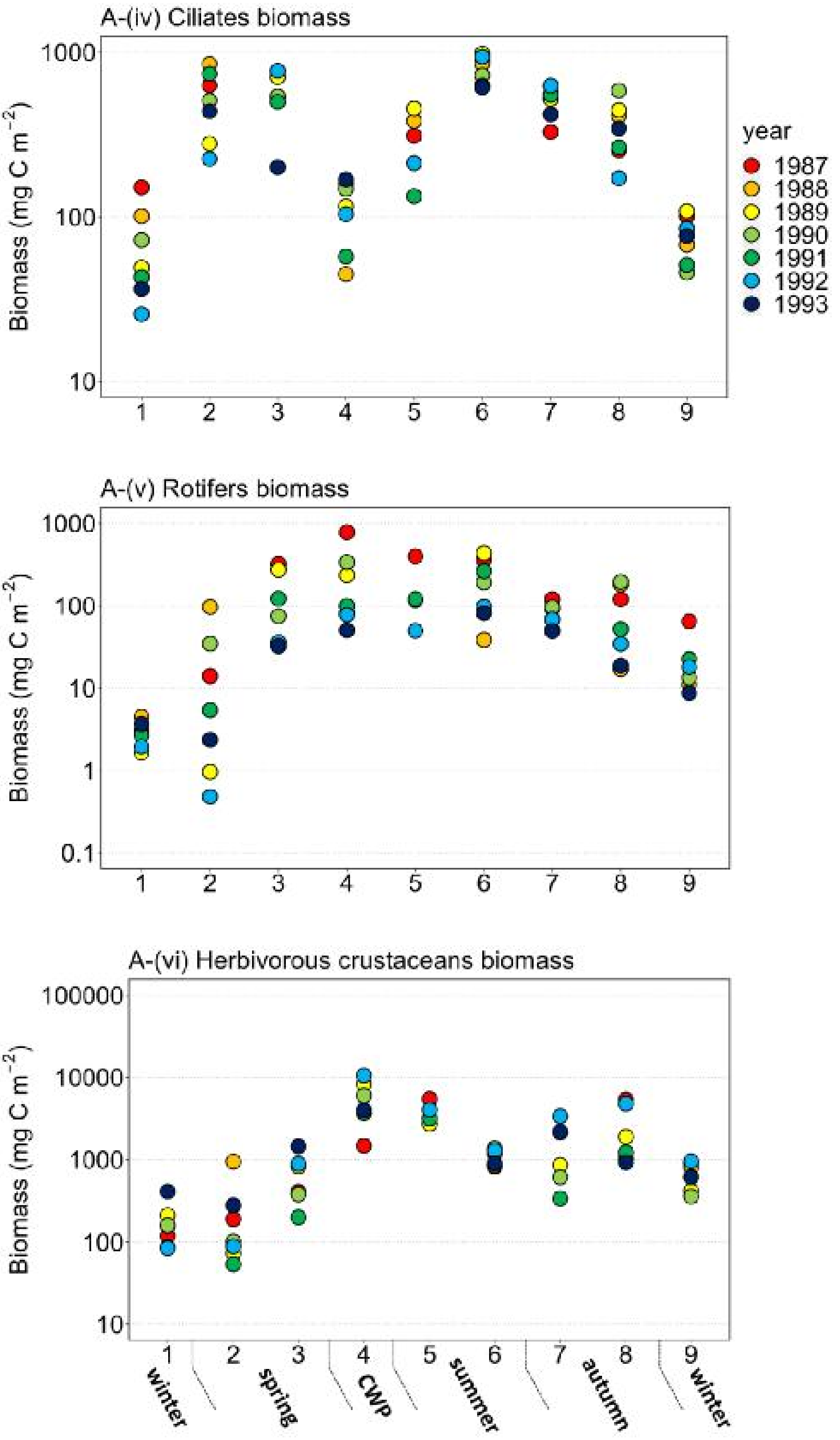
A-(iv) Ciliates biomass
Biomass (mg C m^{-2})
1000
100
10
1 2 3 4 5 6 7 8 9
year
1987
1988
1989
1990
1991
1992
1993
A-(v) Rotifers biomass
Biomass (mg C m^{-2})
1000
100
10
1
0.1
1 2 3 4 5 6 7 8 9
A-(vi) Herbivorous crustaceans biomass
Biomass (mg C m^{-2})
100000
10000
1000
100
10
1 2 3 4 5 6 7 8 9
winter
spring
CWP
summer
autumn
winter

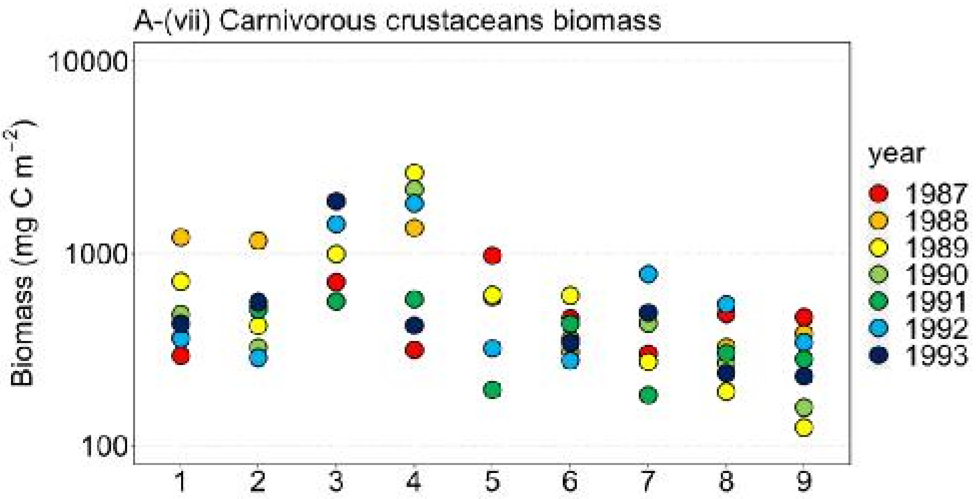
A-(vii) Carnivorous crustaceans biomass
Biomass (mg C m⁻²)
10000
1000
100
1
2
3
4
5
6
7
8
9
year
1987
1988
1989
1990
1991
1992
1993

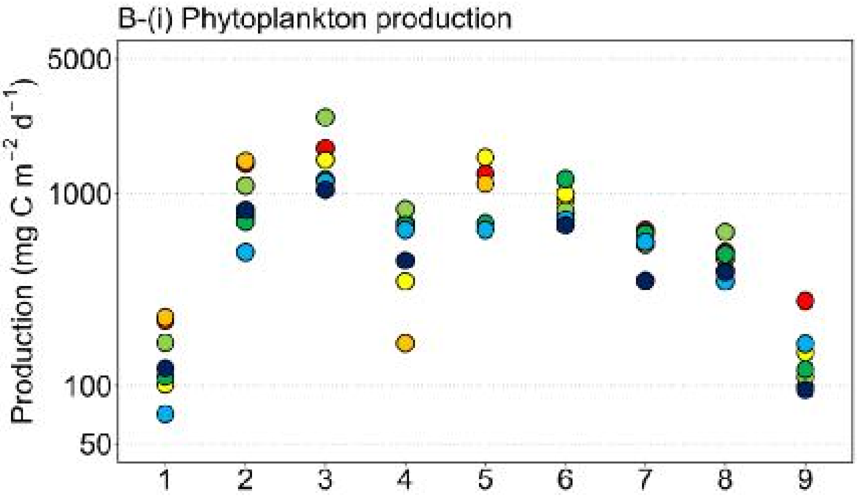
B-(i) Phytoplankton production
Production (mg C m⁻² d⁻¹)
5000
1000
100
50
1
2
3
4
5
6
7
8
9

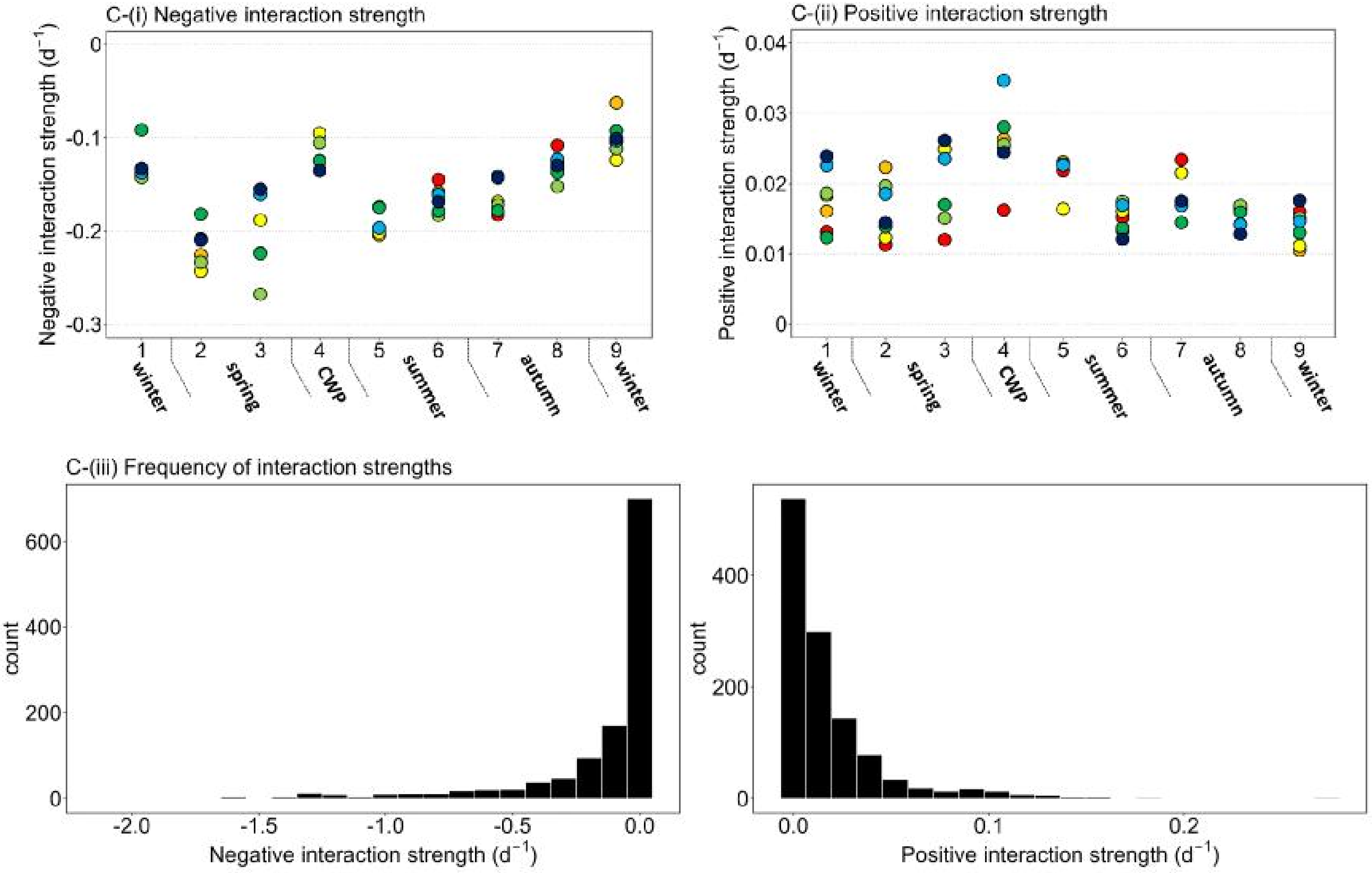
C-(i) Negative interaction strength
Negative interaction strength (d^{-1})
0
-0.1
-0.2
-0.3
1 2 3 4 5 6 7 8 9
winter
spring
CWP
summer
autumn
winter
C-(ii) Positive interaction strength
Positive interaction strength (d^{-1})
0.04
0.03
0.02
0.01
0
1 2 3 4 5 6 7 8 9
winter
spring
CWP
summer
autumn
winter
C-(iii) Frequency of interaction strengths
count
600
400
200
0
-2.0
-1.5
-1.0
-0.5
0.0
Negative interaction strength (d^{-1})
count
400
200
0
0.0
0.1
0.2
Positive interaction strength (d^{-1})

Appendix Figure 2

A. *Seasonal dynamics in biomasses of (i) phytoplankton, (ii) bacteria, (iii) heterotrophic flagellates, (iv) ciliates, (v) rotifers, (vi) herbivorous crustaceans and (vii) carnivorous crustaceans for each year and phase (all in mg C m^{-2}).*

B. *Seasonal dynamics in (i) net primary production for each year and phase (mg C m^{-2} d^{-1}).*

C. *Seasonal dynamics in (i) negative interaction strengths and (ii) positive interaction strength for each year and phase (d^{-1}). They are averages of the strength of all interactions in a particular phase and year. (iii) Frequency distribution of the negative and positive interaction strengths.*

Please note that

· Biomasses and total fluxes are plotted on different $\log_{10}$ scales. Biomasses of most trophic guilds reached maximum values during spring and summer, caused by favourable growth conditions (light, temperature, partly nutrient availability). During the clear-water phase (CWP), the smaller guilds, i.e., phytoplankton, flagellates and ciliates, showed temporary decreases in biomass caused by competition and grazing by the larger guilds, i.e., crustaceans, which reach their maximum biomasses around the CWP.

· The net primary production (which is a very strong predictor for the total food web flux) reached the highest values during spring and summer, with a temporary decrease during the clear-water phase due to low algal biomass. Phytoplankton production contributed 69% of the total food web flux, bacterial production 9% and the production of the other guilds between 1 and 7% each.

· The seasonal pattern of the negative interaction strengths reflects the bimodal pattern found in the biomasses of the small, metabolically most active guilds, i.e., phytoplankton, flagellates and ciliates, and in primary production with high values during spring and summer and a temporary decrease during the CWP; this pattern was less pronounced in the positive interaction strengths. The negative interaction strengths are much larger than the positive interaction strengths, because in positive interaction strengths an efficiency (<1) is present (Equations (1) and (2)). This is in agreement with earlier theoretical arguments (Pimm & Lawton 1977; Yodzis 1981).

· The interaction strengths were strongly skewed with a majority of weak interactions. Considering the frequency distribution accounting for all phases and years (Appendix Fig. 3 C (iii)) the skewness values were -2.88 for the negative interaction strengths and +3.49 for the positive interaction strengths. Both values indicate strong skewness. Similar frequency distributions were found for the Ythan estuary food web (Emmerson & Raffaelli 2004). There were no large differences in skewness between the phases (combining all years) with skewness values ranging from -3.44 to -2.10 for the negative interaction

strengths (average: -2.86; standard deviation 0.40), and from 2.07 to 3.37 for the positive interaction strengths (average: 2.52; standard deviation 0.42).

Appendix S3 Underlying assumptions of the loop weight approach and their potential bearing on our results

We used the loop weight approach to examine the linear, asymptotic stability of food webs. We restricted the analysis to positive loops of length 3 because in 56 out of the 59 webs the loop with the maximum weight had a length 3. For the three other webs, the overall maximum loop weight was close to the maximum weight of the loops with length 3. Furthermore, Neutel et al. (2002) suggested that maximum loop weight of loops of length 3 is the best indicator for stability. We could derive loop weights directly from the data but not all assumptions underlying the loop weight approach were fulfilled.

The first assumption is that the food webs are in equilibrium, i.e., that there is no net change of the biomasses of the guilds over time. The equilibrium assumption is required for the derivation of interaction strengths. It was obviously not satisfied, given the seasonal biomass dynamics in Lake Constance. However, we accounted for biomass changes between phases when calculating the mass-balanced food webs and thus interaction strengths to correct for imbalances between inflow (gross primary production or ingestion) and outflow (losses by excretion, respiration, predation or sedimentation) of a guild (Gaedke et al. 2002; Hart et al. 1997, see **Appendix Fig. 3.1**).

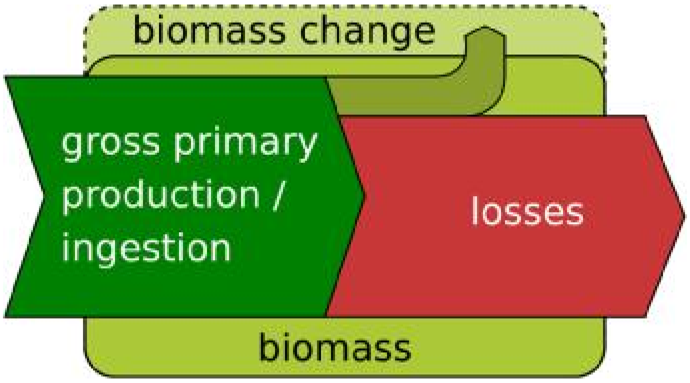

***Appendix Fig. 3.1**: Recorded temporal changes in the biomass of a guild between phases were used to correct for a mismatch between gross primary production or ingestion and loss processes of a guild and thus to prevent over- or underestimating a flow entering or leaving a guild in the mass-balanced food web.*

The equilibrium assumption is also central to the concept of asymptotic stability (and thus, by extension, for the loop weight approach), which defines stability as whether or not a system returns to an equilibrium point when slightly perturbed away from it. As a first indication that the food webs we considered are close to such an equilibrium, we consider the daily net rate of change of phytoplankton biomass between phases 1 and 2 in 1990. We picked this case as a conservative example because the increase of phytoplankton biomass was particularly large between these two phases (cf. **Appendix Fig. 2A**) and because phytoplankton was always among the guilds that were most decisive for food-web stability (**Appendix Table S1**). In 1990, in phase 1 mean phytoplankton biomass was 261 mg C m^{-2} and in phase 2 it was 1200 mg C

m^{-2}, which amounts to a net growth rate of approximately 17 mg C m^{-2} d^{-1}. This is between 9 and 62 times less than the mean net phytoplankton production rates in these phases (158 mg C m^{-2} d^{-1} and 1051 mg C m^{-2} d^{-1}, respectively).

More precisely, the linear approximation of the biomass dynamics of all guilds, dX/dt, with X_i being the biomass of the i-th guild, around some specific system state $\tilde{X}$ is given by

$$\frac{dX}{dt} \approx F(\tilde{X}) + J(\tilde{X}) \cdot (X - \tilde{X}), \qquad \text{(Eq. S3.1)}$$

where $F(\tilde{X})$ are the (measured) net growth rates at state $\tilde{X}$, $J(\tilde{X})$ is the Jacobian matrix that consists of the measured interaction strengths at $\tilde{X}$, and $(X - \tilde{X})$ is a (usually) small perturbation away from $\tilde{X}$. If $\tilde{X}$ was an equilibrium, the net growth rates $F(\tilde{X})$ would be zero and only in this case *Re*(λ_{max}) of $J(\tilde{X})$ would precisely describe whether the perturbation declines or grows over time (i.e., whether the equilibrium is stable or not). If $\tilde{X}$ is not an equilibrium and thus $F(\tilde{X}) \neq 0$, this first term of the right-hand side of Eq. (S3.1) will – in a strict mathematical sense – dominate the dynamics, irrespective of the relative magnitudes between the net growth rates and interaction strengths, because the perturbation is assumed to be arbitrarily small. However, this does not reflect the actual situation of the empirical food webs we considered. As shown in **Appendix Fig. 2A**, in each phase the biomasses of the guilds varied between the years, and the standard deviation of this scatter can be used as an approximation of the perturbation $(X - \tilde{X})$. Thus, the sum of the standard deviations of the guilds interacting with a focal guild (phytoplankton in our example) multiplied with the corresponding elements of the focal guild's row of the Jacobian matrix yields a more precise reference point to gauge its net growth rate against. For phytoplankton in phase 1 in 1990 we find that the corresponding second term from Eq. (S3.1) is about 4 times greater than its net growth rate. Considering that the example was deliberately chosen to be very conservative (i.e., in most cases, net changes of biomass over time are much smaller), we conclude that the equilibrium viewpoint is indeed justified.

As a side remark, *Re*(λ_{max}) is sometimes presented as a simple, dimensionless number, which neglects that it does in fact carry a unit (the same applies to LW_{max}). We adhered to the approach of May (1973) where the interaction strengths, i.e., the elements of the Jacobian matrix, are calculated by taking the derivatives of the growth rates of the species (unit: mg C m^{-2} d^{-1}) with respect to the biomass densities of the species (unit: mg C m^{-2}) and as such carry the unit d^{-1}. In our case, the off-diagonal elements of the Jacobian simplify to the expressions given in Eqs. (1) and (2), having also the unit d^{-1}. The way *Re*(λ_{max}) and LW_{max} are calculated from these matrix elements ensures that they have the same dimension as the matrix elements and thus the same unit d^{-1}. For the loop weights (and thus LW_{max}) this can be verified using Eq. (3) or (4).

Appendix S4 Seasonal and interannual variability of Jacobian stability ($Re(\lambda_{max})$) and its correlation and temporal synchronisation with maximum loop weight

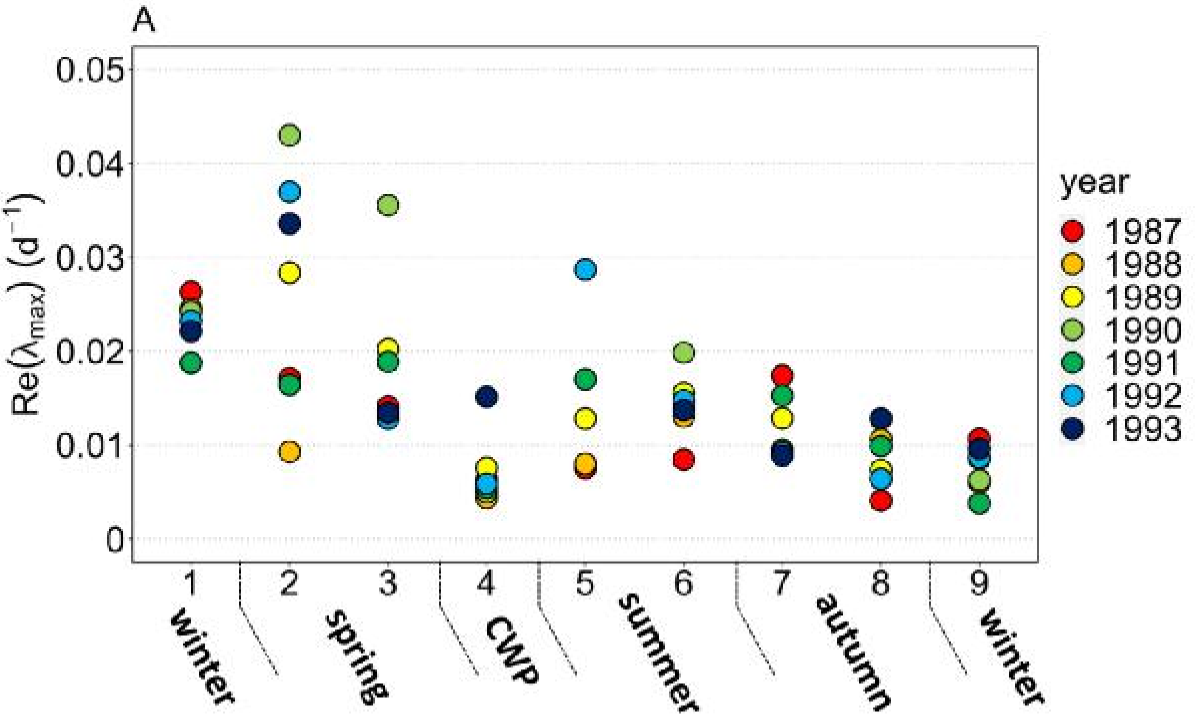

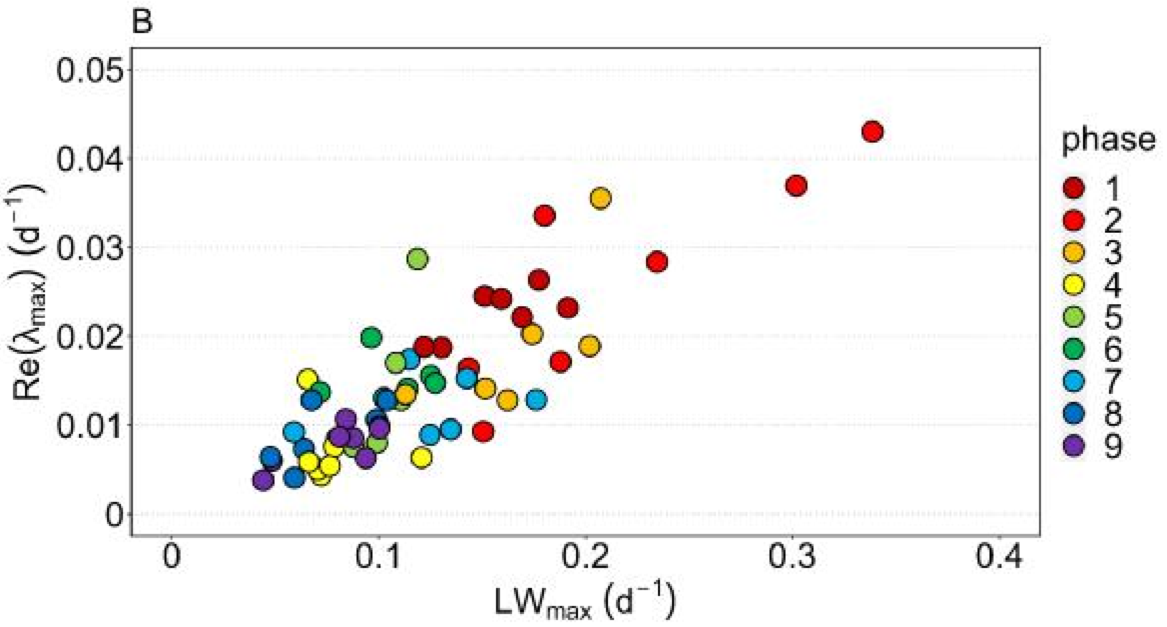

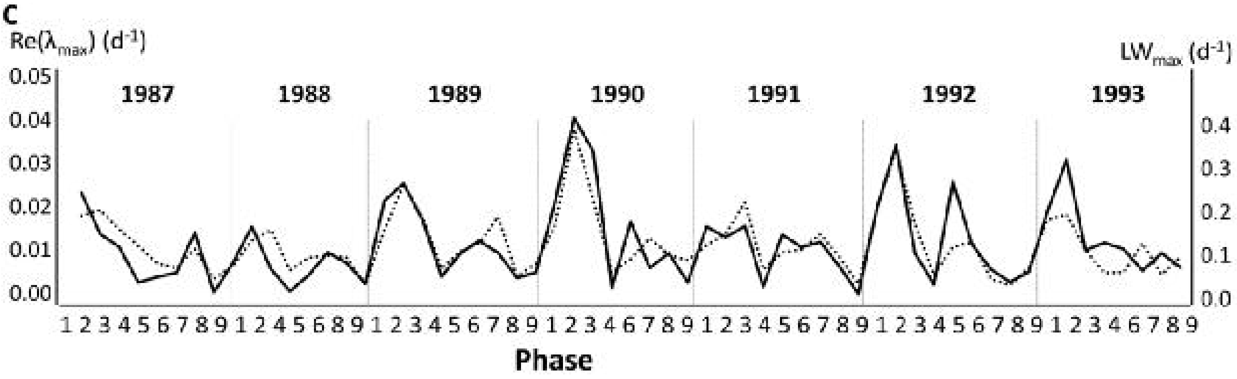

Appendix Figure 4

A. *Jacobian (linear asymptotic) stability (Re(λ_{max})) per phase over all 7 years.*

B. *Spearman correlation between Re(λ_{max}) and LW_{max} (r_S=0.78, p<0.001).*

C. *Long-term dynamics in stability (Re(λ_{max})*, full line*) and maximum loop weight (LW_{max}*, dotted line*) over the seven years of observation. Both measures exhibit similar seasonal and interannual variation.*

Appendix S5 Calculation of the openness at individual guilds and their correlation with interaction strengths

A) Calculation of O_1 – O_4:

For a loop of length 3 starting with the primary resource (**Figure 1B**), here phytoplankton, we distinguished four types of in- and outgoing fluxes. We defined their openness (O_i for flux i (=1,2,3,4)) as the ratio between the sum of all fluxes that go out of (arrows 1 and 3), or in (arrows 2 and 4) a considered trophic guild (G) but are not part of the loop (=F_{G-O})) and the sum of all fluxes at the considered trophic guild . For example, for flux (1) (**Figure 1B**), we considered the five outgoing fluxes (**Figure 1A**), took the total of all fluxes from phytoplankton (P) to guilds outside the loop (F_{P-O}), namely the three fluxes from phytoplankton to rotifers F_{P-R}, to herbivorous crustaceans F_{P-Ch}, and to carnivorous crustaceans F_{P-Cc}, i.e., $F_{P-O} = F_{P-R} + F_{P-Ch} + F_{P-Cc}$. Then we took the two fluxes from phytoplankton within the loop F_{P-L}, i.e., the flux from phytoplankton to flagellates F_{P-F}, and the one to ciliates F_{P-C}, summing these gives $F_{P-L} = F_{P-F} + F_{P-C}$. We then calculate the first type of openness (O_1) as the ratio:

$$O_1 = \frac{F_{P-O}}{F_{P-O}+F_{P-L}},$$

In analogy,

$$O_2 = \frac{F_{O-F}}{F_{O-F}+F_{P-F}}$$

being the fraction of the diet the flagellates get from outside the loop compared to the total food intake,

$$O_3 = \frac{F_{F-O}}{F_{F-O}+F_{F-C}}$$

being the fraction of the production of the flagellates which leaves the loop compared to its total production, and

$$O_4 = \frac{F_{O-C}}{F_{O-C}+F_{P-C}+F_{F-C}}$$

being the fraction of the diet of the ciliates coming from outside the loop compared to the total ingestion of the ciliates.

B) Correlation between O_1 – O_4 and interaction strengths

According to expectations (cf. main text), O_i was related to particular fluxes in the loop and thus the corresponding interaction strengths. To quantify these relationships, we correlated O_i with the absolute values of the respective interaction strengths. It appeared that:

- O_1 was negatively correlated with the strength of the negative effect of flagellates on phytoplankton (r_S=-0.58) and the strength of the positive effect of phytoplankton on ciliates (r_S= -0.59).
- O_2 was negatively correlated with the strength of the negative effect of flagellates on phytoplankton (r_S=-0.83).
- O_3 was negatively correlated with the strength of the negative effect of ciliates on flagellates (r_S=-0.66).
- O_4 was - weakly - negatively correlated with the strength of the positive effect of phytoplankton on ciliates (r_S=-0.26) and correlated with the strength of the negative effect of ciliates on flagellates (r_S=-0.68).

Thus, O_i was slightly more strongly correlated with overall loop weight, $LW_{C\text{-}F\text{-}P}$, than with the interaction strengths in which the particular guilds were involved. This can be explained by the fact that the different types of openness O_i and interaction strengths in the loop are not independent from each other and reinforced their effect on $LW_{C\text{-}F\text{-}P}$, e.g., O_1 and O_3 both depend on the intensity of grazing by crustaceans, which varied strongly across the season.

Appendix S6 Relationship between the interannual diversity in the heaviest loops and stability

To test for a potential diversity effect on stability we considered the number and frequency of the different loops which became the heaviest during the individual phases which varied from one (phase 1, only the ciliates-flagellates-phytoplankton loop), two (phases 2, 3, 7, 8 and 9), three (phases 5 and 6), and up to four (phase 4) during the 7 years of investigation (cf. **Appendix Table S1**). We used this measure of diversity as an indicator for the flexibility of the food web to adjust to ambient conditions. We accounted for the number of different loops and their evenness, which were the heaviest during the individual phases. For example, in phase 2 two different loops became the heaviest (cf. **Appendix Table S1**), one of them in 5 out of 7 years (p_i = 5/7=0.71) and one in 2 of the 7 years (p_i = 2/7=0.29). We used the Simpson index D which was calculated as $D = 1 – \Sigma p_i^2 = 0.59$. A seasonal pattern similar to the one established for D was found when using the Shannon-Wiener index H`.

Appendix Fig. 6 reveals an overall positive covariation between loop diversity and stability with low diversity and stability (i.e., high values of 1-D, $Re(\lambda_{max})$ and LW_{max}) during late winter/spring, minimum values during the clear-water phase and a re-increase thereafter. During late summer, autumn and winter the system is more stable than expected from diversity compared to spring and early summer. This might be attributed to the relatively low primary production and temperature reducing the metabolic rates and thus loop weight.

The high interannual diversity in the type of the heaviest loop during and to a lesser extent also after the clear-water phase (phase 4, 5 and 6) is in line with an earlier study revealing that consistently across the study period a clear-water phase with low phytoplankton biomass due to severe grazing pressure did occur in Lake Constance in late May/June despite pronounced variability in weather conditions during late winter and spring. However, which phytoplankton grazers initiated the clear-water phase varied substantially among years depending on the winter/spring weather conditions (Tirok & Gaedke 2006). Our study suggests that the diversity in the functionally different types of grazers (i.e., ciliates, rotifers, herbivorous and carnivorous crustaceans) present around the time of the clear-water phase in May/June provides not only a buffering mechanism in respect to the seasonal recurrence of the plankton dynamics but also enhances food web stability.

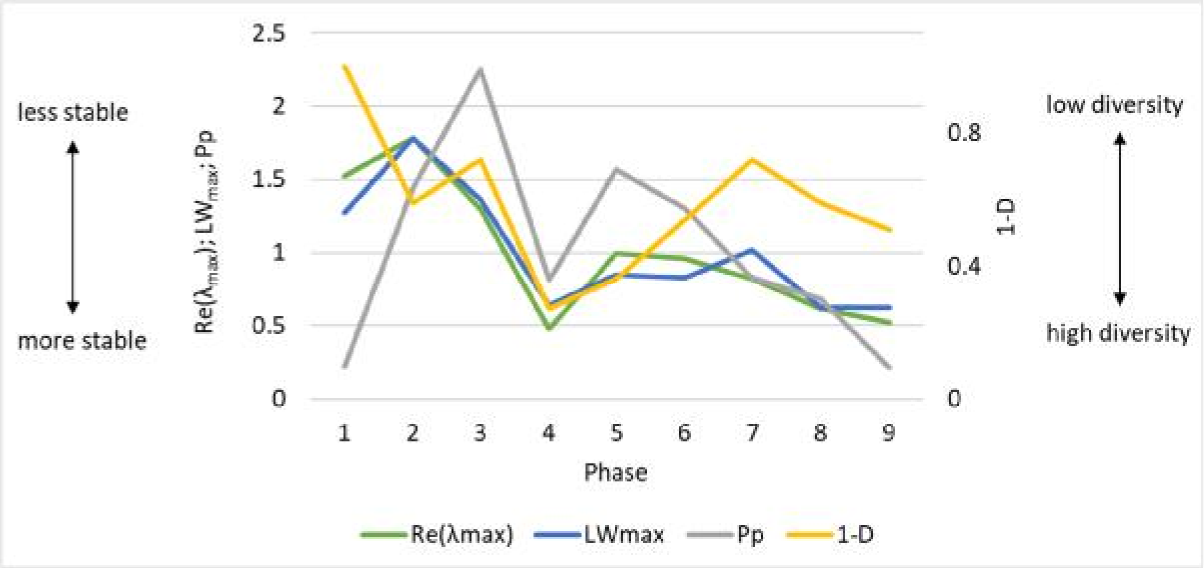

Appendix Figure 6 *Standardized time series of the Jacobian (linear asymptotic) stability Re(λ_{max}), LW$_{max}$, and phytoplankton production Pp (values for each phase are averages across years divided by the long-term mean across the 59 webs (left y-axis)) and of the diversity in the loops which became the heaviest during a distinct phase using the Simpson index D (right y-axis). The values displayed are 1-D, i.e., low values on both y-axes indicate high stability and diversity, respectively. High values on the left y-axis indicate high production.*

Appendix S7 Relationship between overall openness and loop weight for the six loops, which were temporally the heaviest ones.

(A)

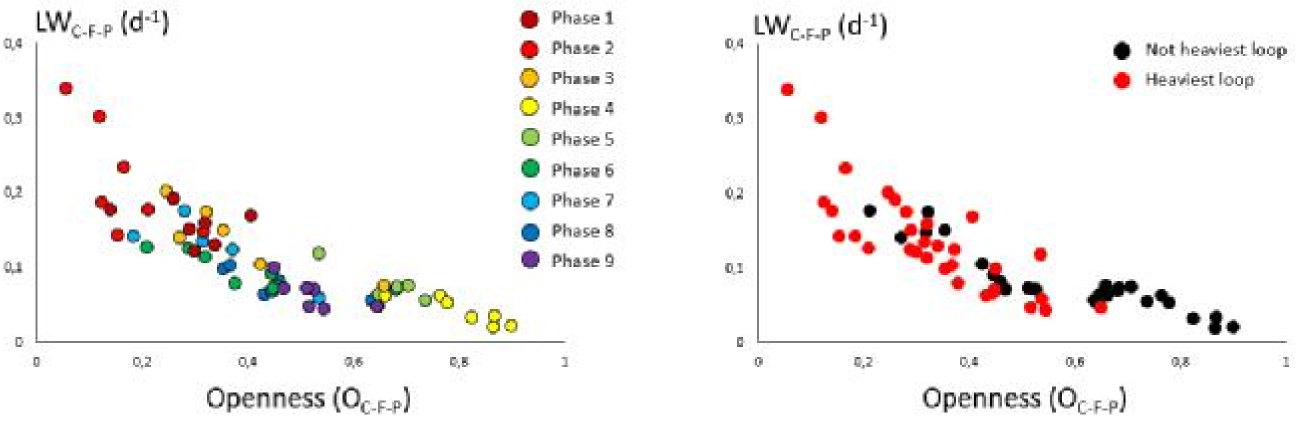

(B)

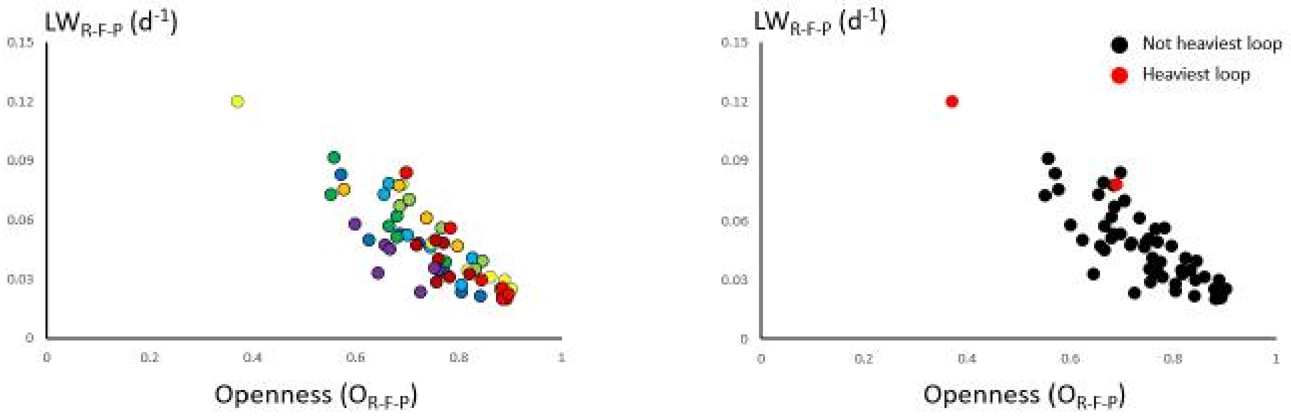

(C)

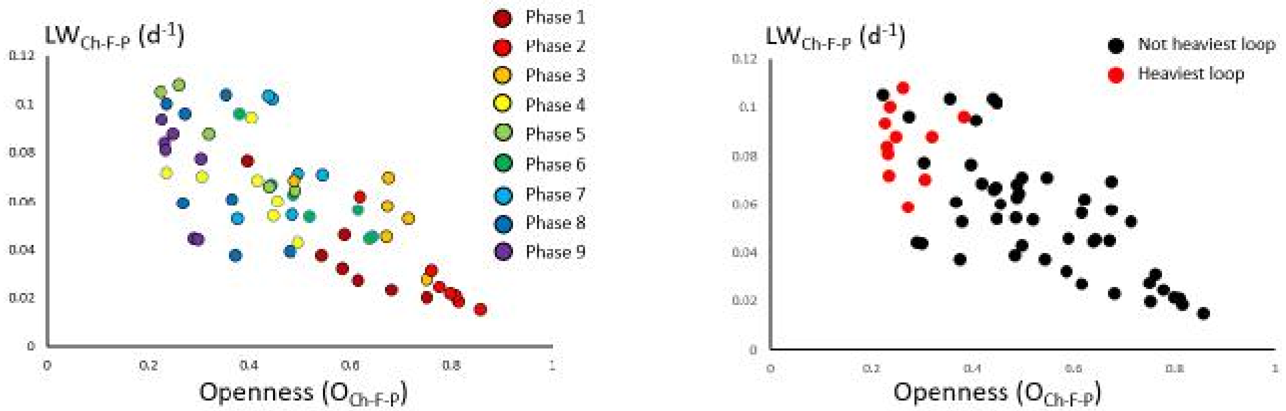

(D)

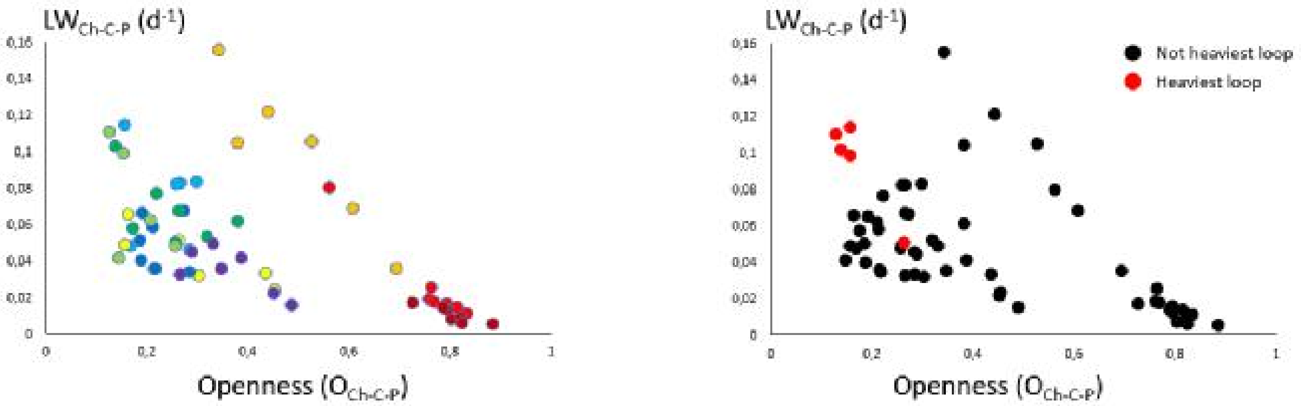

(E)

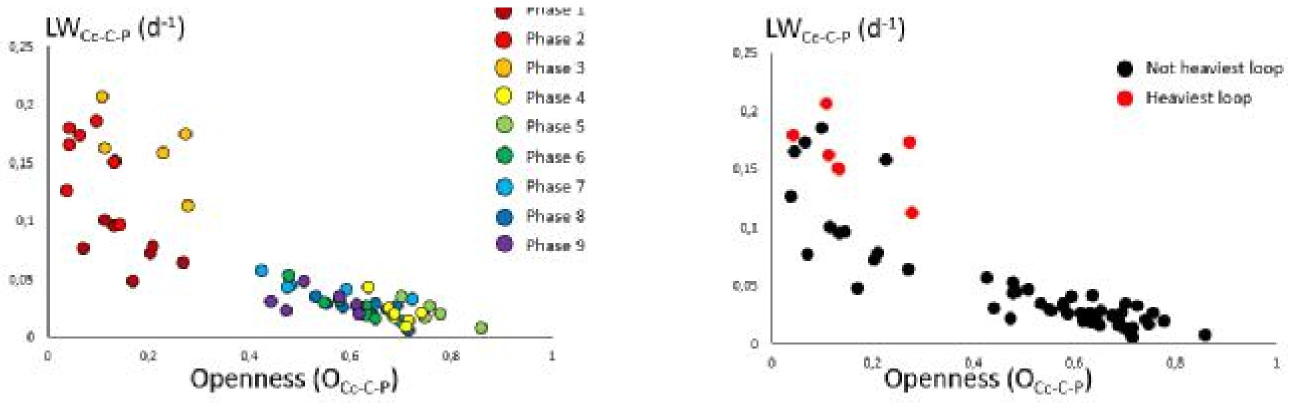

(F)

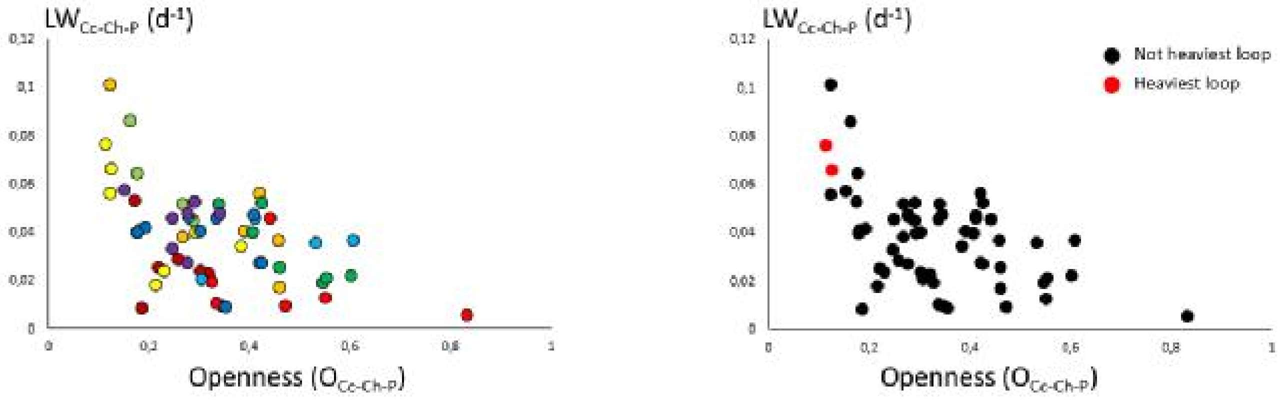

***Appendix Figure 7.1** Relationship between loop weight and overall openness O_O **A)** ciliates-flagellates-phytoplankton loop (Spearman correlation r_S = -0.90), **B)** rotifers-flagellates-phytoplankton loop r_S = -0.81, **C)** herbivorous crustaceans-flagellates-phytoplankton loop r_S = -0.73, **D)** herbivorous crustaceans-ciliates-phytoplankton loop r_S = -0.62, **E)** carnivorous crustaceans-ciliates-phytoplankton loop r_S = -0.88, and **F)** carnivorous crustaceans-herbivorous crustaceans-phytoplankton loop r_S = -0.48. The left panels provide the phase and*

the right panels whether (red dots) or not (black dots) the loop was the heaviest during that phase and year.

Overall openness explains a large fraction in the seasonal and interannual variability of the loop weight of all loops that became the heaviest during distinct phases. This confirms the results presented for the ciliates-flagellates-phytoplankton loop presented in the main text. In principal, it is not unexpected that there is a negative correlation between overall openness and the weight of the respective loops. After all, a loop cannot be very open and very heavy at the same time, explaining why the upper right triangle in the panels of **Appendix Fig. 7.1** is always empty. However, in several loops (most notably C-F-P, R-F-P and Ch-F-P) the relation between openness and loop weight followed a relatively narrow band, i.e., when these loops were closed, they tended to be heavy (for a reason for a deviation from this band in some loops see **Appendix S8**). To test how much of this relation was driven by ecological constraints instead of mathematical inevitability, we randomized the food webs by permuting the fluxes (100 random permutations for each of the 59 food webs) but kept the link structure (i.e., who eats whom) and biomass densities of the groups fixed. We then calculated the overall openness and the loop weight of the C-F-P loop in these randomized food webs. We found the correlation between them to still exist (as expected the upper right triangle is empty), but to be considerably weakened (Spearman correlation r_S = -0.75 in the randomized webs vs. r_S = -0.90 in the empirical webs, **Appendix Fig. 7.2**). Notably, in many webs loop weight is low despite low openness.

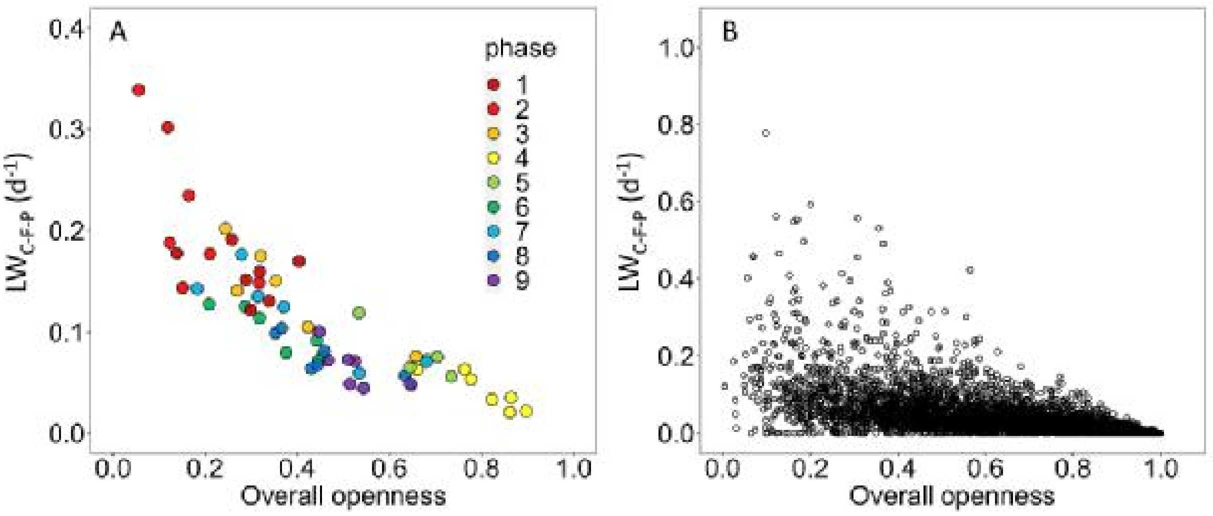

Appendix Figure 7.2 *Openness vs. loop weight of the C-F-P loop in the empirical (A, Spearman correlation r_S = -0.9) and randomised food webs (B, Spearman correlation r_s = -0.75).*

Appendix S8 Impact of the distribution of fluxes within the loop on loop weight

The relationship between overall openness and loop weight also depended on the structure of the fluxes within the loop. In the case that the majority of the production of the primary resource is consumed by the intermediate consumer, which, in turn, provides most of the energy to the top predator, the fluxes F_{bi} and F_{it} are relatively high. This implies that the two negative interaction strengths are comparably high, resulting in a relatively high loop weight at a given openness. In contrast, if most of the primary resource production is directly consumed by the top predator, then F_{bt} is large, and only the positive interaction strength will be promoted whereas the two negative interaction strengths are relatively weak which on overall will reduce loop weight. This effect on loop weight can be clearly seen when considering the herbivorous crustacean-ciliates-phytoplankton loop (**Appendix Figure 7D**). During phase 3, most of the primary production is consumed by ciliates, representing the intermediate consumer (cf. **Appendix Figure 1C**), and loop weight is already very high at an intermediate openness (**Appendix Figure 8 below**). In contrast, in phase 4 the herbivorous crustaceans as top predators dominate (cf. **Appendix Figure 1D**) and loop weight is much lower at a similar openness.

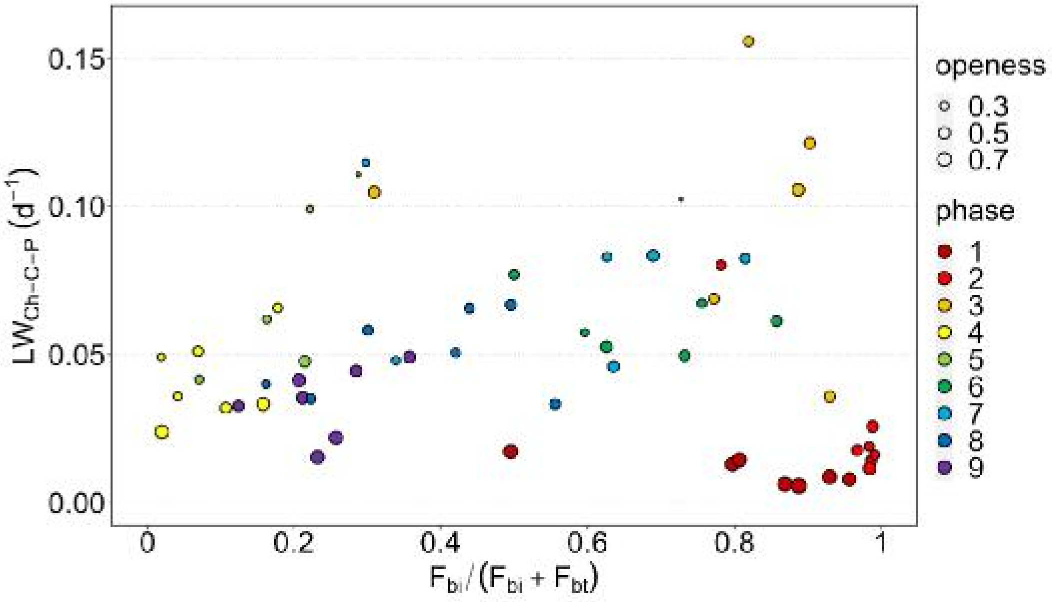

Appendix Figure 8 *Relationship between the loop weight of the herbivorous crustacean-ciliates-phytoplankton loop and the fraction of the primary production, which is consumed by ciliates, i.e., the intermediate consumer, relative to the total primary production consumed in the loop. The size of the circles continuously increases with openness.*

Appendix S9 Energetic constraints within loops restrict maximum loop weight in mass-balanced food webs

Li et al. (2021) revealed that in real food webs loop weight is restricted by energetic constraints imposed on individual loops. The analysis was based on simulation results and one empirical food web. Such constraints are not found in random webs. The constraints arise from the metabolic properties of the trophic guilds and mass-balanced constraints. For example, since the production of the primary resource can only be consumed once, a high flux from this guild directly to the top predator implies that the fluxes to the intermediate consumer, and that from the intermediate consumer to the top predator have to be small. Furthermore, the fluxes from the primary resource via the intermediate consumer to the top predator and the corresponding interaction strengths can only be high if the production of the primary resource is high. However, this requires a sufficiently high biomass of the primary resource, which lowers the ratio between the flux directly to the top predator and its biomass and hence the positive interaction strength (cf. eq. 3). In addition, the biomasses of the intermediate and top consumer tend to be negatively correlated as they compete for the primary resource production, which promotes a negative correlation between the two negative interaction strengths (for further mechanisms see Li et al. 2021).

The Lake Constance dataset confirms these results, revealing compensatory effects that mitigate loop weight, thereby enhancing food web stability. Within the 25 positive feedback loops of the Lake Constance food web, each comprising two top-down-based negative interactions and one bottom-up-based positive interaction, strong positive interactions generally coincided with weak negative interaction strengths and vice versa, resulting in low loop weight (**Appendix Figure 9.1A**). None of the 25 x 59 = 1475 loops examined had simultaneously high negative and positive interaction strengths and in most loops none or either the positive interaction or the product of the two negative ones was large. Loop weight was relatively high when all interaction strengths were moderately high. Thus, the inevitable energetic constraints within individual loops clearly restrict maximum loop weight. In line, the efficiency of this compensatory mechanism among interaction strengths had a strong impact on loop weight (**Appendix Figure 9.1B**).

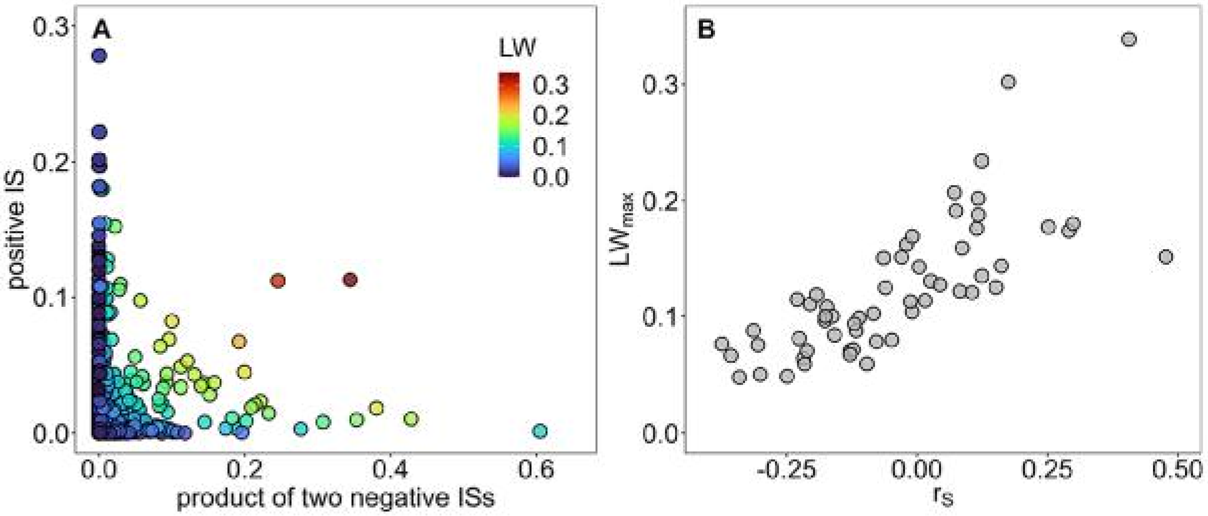

Figure 9.1 (A) *Relationship between the product of the two negative interaction strengths (ISs) and the one positive interaction strength across the 25 positive feedback loops of the food web of Lake Constance over the different years and phases, i.e., 59 webs. Colours denote loop weight (LW).* ***(B)*** *Relationship between the Spearman correlation coefficient (r_S) between the two negative interaction strengths and the one positive interaction strength within the 25 positive feedback loops, for a specific year and phase, and the corresponding maximum loop weight (LW_{max}).*

Examining the different phases separately reveals that the effectiveness of the compensatory mechanism among positive and negative interaction strengths differed between phases which elucidates why certain phases (e.g., phases 2 and 3) exhibited higher loop weights compared to others. This was attributed to a lower buffering of positive and negative interaction strengths within the heaviest loops, and thus loop weights (**Appendix Figure 9.2**).

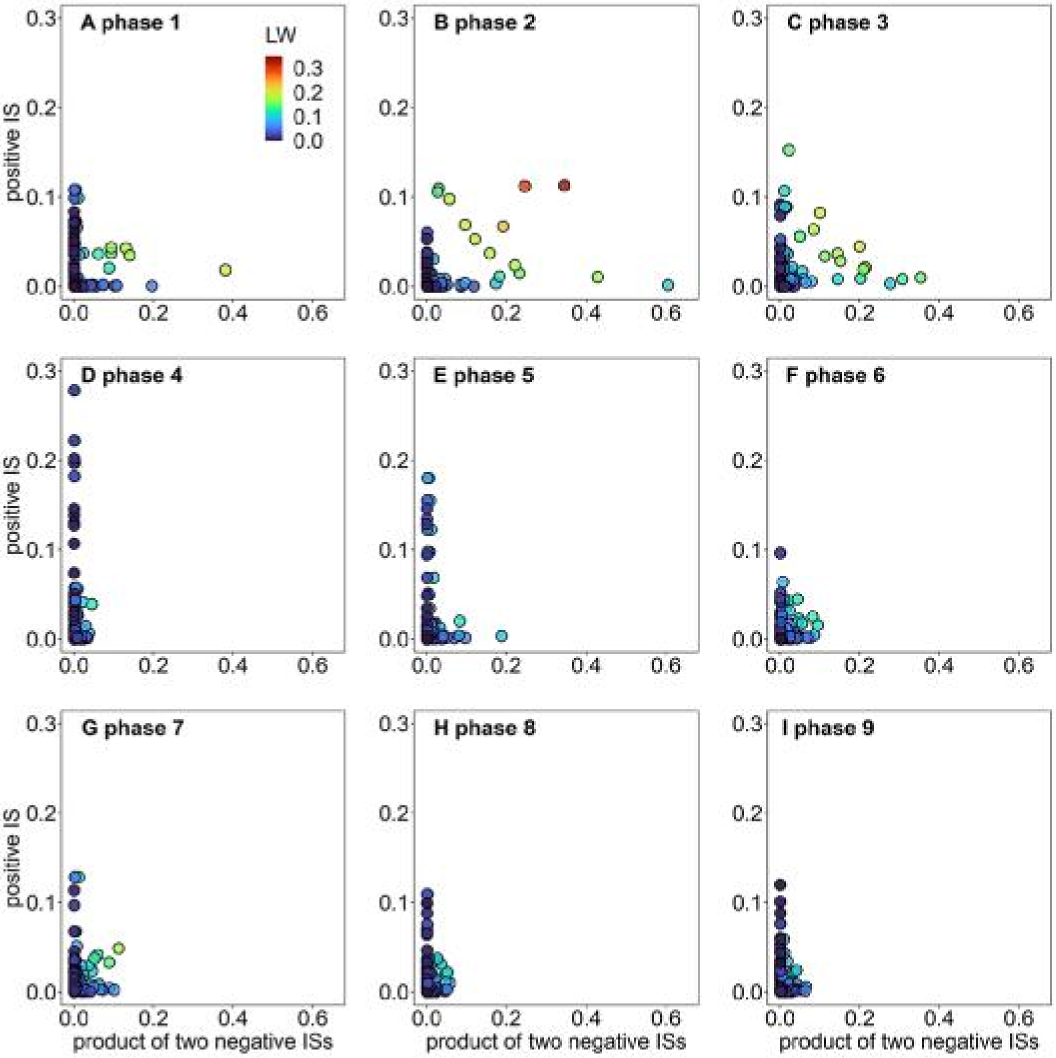

Appendix Figure 9.2 *Relationships between the product of the two negative interaction strengths and the one positive interaction strength within the 25 positive feedback loops across the different phases. Colors denote the loop weight (LW).*

References

Boit, A. & Gaedke, U. (2014). Benchmarking successional progress in a quantitative food web. PLoS One, 9, e90404.

Emmerson, M.C. & Raffaelli, D. (2004). Predator–prey body size, interaction strength and the stability of a real food web. Journal of Animal Ecology, 73, 399–409.

Gaedke, U., Hochstadter, S. & Straile, D. (2002). Interplay between energy limitation and nutritional deficiency: Empirical data and food web models. Ecol Monogr, 72, 251.

Hart, D., Stone, L., Stern, A., Straile, D. & Gaedke, U. (1997). Methods for constructing and balancing ecosystem flux charts: new techniques and software. Environmental Modeling and Assessment, 2, 23–28.

Li, X., Yang, W., Gaedke, U. & de Ruiter, P.C. (2021). Energetic constraints imposed on trophic interaction strengths enhance resilience in empirical and model food webs. Journal of Animal Ecology, 90, 2065–2076.

Neutel, A.-M., Heesterbeek, J.A.P. & de Ruiter, P.C. (2002). Stability in real food webs: weak links in long loops. Science (1979), 296, 1120–1123.

Pimm, S.L. & Lawton, J.H. (1977). Number of trophic levels in ecological communities. Nature, 268, 329–331.

Straile, D. (1998). Biomass allocation and carbon flow in the pelagic food web of Lake Constance. In: Archives Hydrobiologia Special Issue: Advances in Limnology. pp. 545–563.

Tirok, K. & Gaedke, U. (2006). Spring weather determines the relative importance of ciliates, rotifers and crustaceans for the initiation of the clear-water phase in a large, deep lake. J Plankton Res, 28, 361–373.

Yodzis, P. (1981). The stability of real ecosystems. Nature, 289, 674–676.
